# The NF-κB-TNFAIP3 axis attenuates MASLD via activation of hepatocyte autophagy

**DOI:** 10.1038/s41420-026-03310-9

**Published:** 2026-08-25

**Authors:** Qun Sun, Guixin Li, Huixiang Yao, Xiangwei Liao, Long Wang, Jinghui Guo

**Affiliations:** Department of Gastroenterology, Shanghai Sixth People’s Hospital Affiliated to Shanghai Jiao Tong University School of Medicine, Shanghai, China

**Keywords:** Non-alcoholic steatohepatitis, Macroautophagy

## Abstract

Tumor necrosis factor α-induced protein 3 (TNFAIP3) has been implicated in various liver diseases, but its role in metabolic dysfunction-associated steatotic liver disease (MASLD) remains unclear. We analyzed TNFAIP3 expression in diet-induced MASLD mouse models using public transcriptomic data. In vitro, we investigated the effects of TNFAIP3 overexpression and knockdown in HepG2 cells on lipid accumulation, autophagy, and key metabolic pathways. In vivo, a high-fat diet-induced mouse model was used to validate the protective role of TNFAIP3. TNFAIP3 was consistently upregulated in the livers of various MASLD mouse. Overexpression of TNFAIP3 in HepG2 cells reduced lipid droplet accumulation, whereas knockdown aggravated lipid deposition. TNFAIP3 enhanced autophagy by stabilizing ATG4B, and this effect was autophagy-dependent. In vivo, TNFAIP3 overexpression alleviated hepatic steatosis, improved insulin sensitivity, and modulated lipid metabolism-associated pathways. These effects were reversed by autophagy inhibitors. Furthermore, TNFAIP3 upregulation was driven by NF-κB signaling, as inhibition of NF-κB decreased TNFAIP3 levels. The NF-κB–TNFAIP3 axis regulates hepatic lipid metabolism and protects against liver injury in MASLD, primarily through autophagy. These findings suggest that TNFAIP3 could serve as a potential therapeutic target for MASLD and other metabolic liver diseases.

**Figure:**
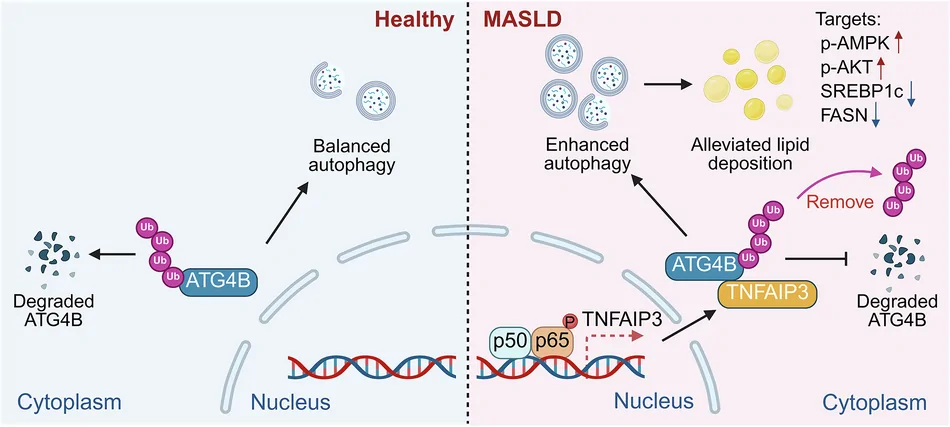

## Introduction

With the rising prevalence of obesity and diabetes, the global incidence of metabolic dysfunction-associated steatotic liver disease (MASLD), also increasing referred to as metabolic dysfunction-associated fatty liver disease nowadays, has reached 38%, making it a vital public health issue [1]. Although MASLD typically progresses slowly, recurrent metabolic dysfunction-associated steatohepatitis (MASH) can accelerate the transition from simple fatty liver to advanced liver disease. It has been reported that ~20% of MASH patients can further develop into liver fibrosis or even hepatocellular carcinoma [2].

It is widely acknowledged that in the context of MASLD, the excessive accumulation of bioactive lipids, such as free fatty acids and cholesterol, within hepatocytes triggers lipotoxicity, which sequentially induces oxidative stress, endoplasmic reticulum stress, and inflammatory cascades [2, 3]. Tumor necrosis factor α-induced proteins (TNFAIPs) have recently been recognized as key regulators of inflammation, immune responses, and tumorigenesis. Among these, TNFAIP3, as a dual-ubiquitin-editing enzyme, comprises an N-terminal ovarian tumor (OTU) domain and seven C-terminal zinc finger domains [4]. The OTU domain possesses deubiquitylase activity, whereas the zinc finger region facilitates protein-protein interactions and self-oligomerizatio, primarily through ubiquitin-binding capabilities [4, 5].

Emerging evidence indicates that TNFAIP3 expression is markedly upregulated in the livers of MASH patients [6, 7]. This finding is recapitulated in preclinical models, where methionine-choline-deficient (MCD) diet-fed mice exhibit elevated hepatic TNFAIP3 protein levels compared to controls [6]. Mechanistic investigations have revealed that TNFAIP3 exerts dual protective functions in hepatocytes: it promotes fatty acid β-oxidation while suppressing fatty acid synthase (FASN) transcription, thereby reducing lipid accumulation; concurrently, it mitigates inflammation by attenuating nuclear factor kappa B (NF-κB) activation in vitro [6, 8]. Additionally, TNFAIP3 directly engages with apoptosis signal-regulating kinase 1 (ASK1) in hepatocytes, deubiquitinating and inhibiting its activation, thereby impeding the progression of MASLD [5].

Macroautophagy (hereafter referred to as autophagy) represents the canonical form of autophagic degradation, whereby specialized cytoplasmic vesicles capture and degrade cellular components via lysosomal enzymes to maintain cellular homeostasis [9]. Orchestrated by autophagy-related genes (ATGs), this tightly regulated process unfolds in six sequential stages: initiation, nucleation, expansion, closure, fusion, and degradation [9]. During expansion, microtubule-associated protein 1 light chain 3 (MAP1LC3/LC3) undergoes initial processing by ATG4 to form LC3-I, which is then activated and conjugated with phosphatidylethanolamine to generate membrane-associated LC3-II. Sequestosome 1 (p62), a key autophagic receptor, bridges ubiquitinated proteins to LC3, facilitating their delivery to autophagosomes [9]. Preclinical and clinical evidence has established a critical role for autophagic dysfunction in MASH. Impaired autophagic flux accelerate MASLD progression in mouse models [9] and individuals with severe steatosis [10]. Mechanistic studies further demonstrate that downregulation of ATG4B disrupts autophagy and suppresses mitochondrial oxidation, thereby accelerating MASLD progression [11]. In ATG4B-deficient mice, compromised autophagic function correlates with exacerbated hepatic steatosis, impaired glucose tolerance, and reduced insulin sensitivity [12].

Despite emerging evidence implicating TNFAIP3 in inflammatory and metabolic regulation, its precise role in MASLD pathogenesis remains incompletely understood. In the present study, we demonstrate that TNFAIP3 expression is upregulated via the NF-κB pathway in both palmitic acid (PA)-treated hepatocytes and diet-induced MASLD mouse models. Mechanistically, TNFAIP3 mitigates hepatic lipid accumulation by deubiquitinating ATG4B, thereby promoting autophagic flux. These findings delineate a previously unrecognized NF-κB-TNFAIP3 axis that may serve as a potential therapeutic target for MASLD.

## Results

### TNFAIP3 exhibits consistent upregulation in the livers of various diet-induced MASLD mouse models

**Fig. 1:**
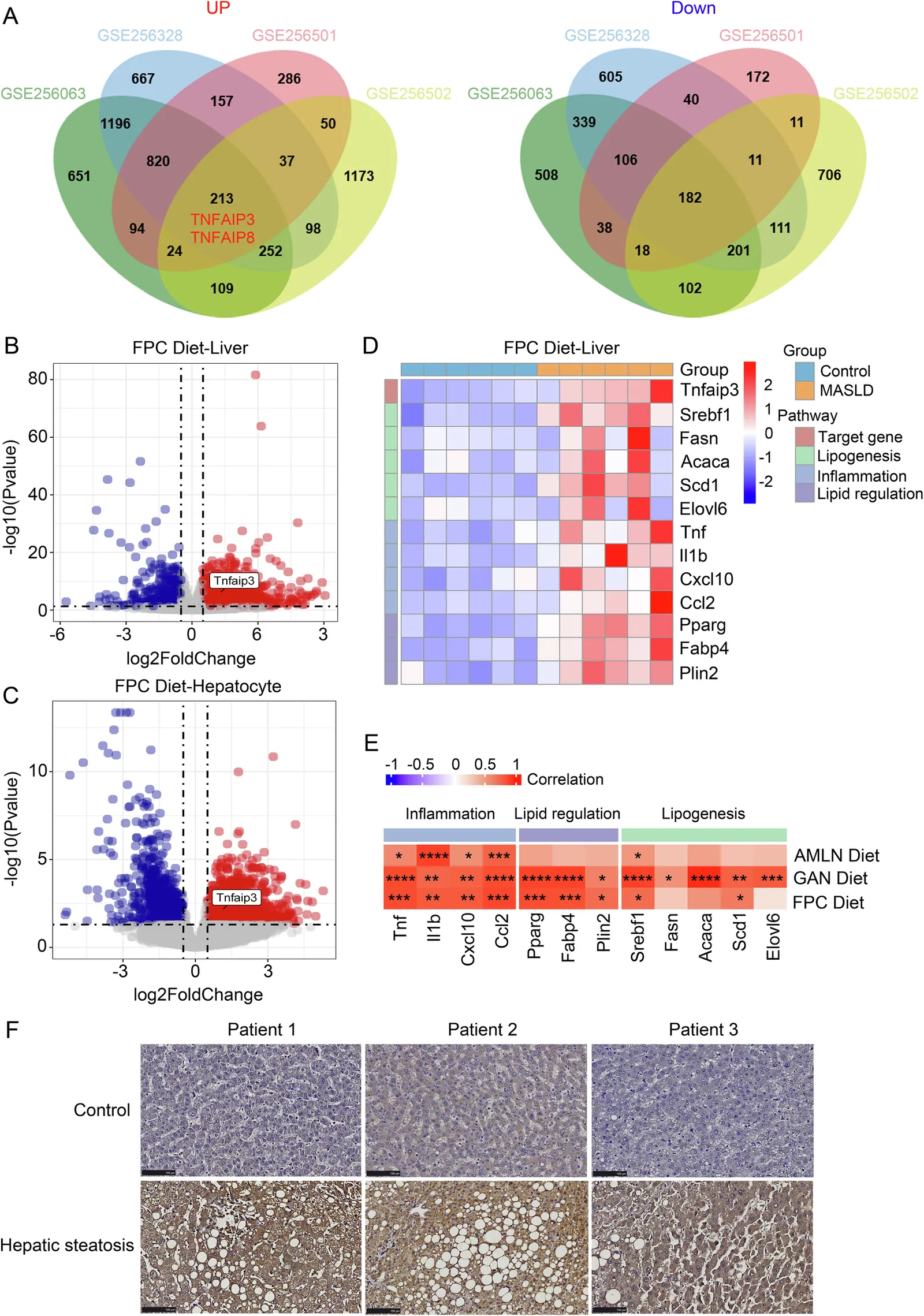
TNFAIP3 is upregulated in the livers of mice with diet-induced MASLD across distinct models. **A** Venn diagram illustrating the number of genes that are commonly upregulated and downregulated in the livers of MASLD mice compared to controls across different datasets. Volcano plots showing differentially expressed genes in the livers (**B**) and isolated hepatocytes (**C**) of mice fed a FPC diet, in comparison with their respective controls. **D** Heatmap presenting the hepatic expression of genes involved in lipogenesis, inflammation, and lipid regulation in mice fed a FPC diet. **E** Correlation heatmap depicting the relationships between TNFAIP3 and genes associated with lipogenesis, inflammation, and lipid regulation across diet-induced MASLD mouse models. **F** Representative immunohistochemical staining of hepatic TNFAIP3 in human livers. *P < 0.05; **P < 0.01; ***P < 0.001; ****P < 0.0001.

To investigate the hepatic expression pattern of TNFAIP family in MASLD, we performed transcriptomic analyses on mouse livers using publicly available data. These datasets corresponded to mice fed with fructose-palmitate-cholesterol (FPC, GSE256501, GSE256502), Amylin liver NASH (AMLN, GSE256063) or Gubra-Amylin NASH (GAN, GSE246328) diets. Notably, among the TNFAIP family members, only TNFAIP3 and TNFAIP8 showed significant upregulation across multiple mouse models (Fig. 1A). When compared to their respective controls, volcano plots demonstrated that TNFAIP3 was significantly upregulated in the livers of all three models (Fig. 1B, Supplementary Fig. S1A), and only TNFAIP3, not TNFAIP8, exhibited more than a two-fold increase in expression across these three datasets. Furthermore, in the FPC diet-induced mouse model, TNFAIP3 expression was also substantially elevated in isolated primary hepatocytes. This finding indicates that the increase in TNFAIP3 occurs specifically within hepatic parenchymal cells rather than being confined to non-parenchymal regions (Fig. 1C).

In addition to TNFAIP3, the expression of genes involved in key metabolic processes was significantly upregulated in the MASLD groups, as shown in Fig. 1D and Supplementary Fig. S1B. Specifically, genes related to lipogenesis (including Srebf1, Fasn, Acaca, Scd1, Elovl6), inflammation (such as Tnf, Il1b, Cxcl10, Ccl2), and lipid regulation (like Pparg, Fabp4, Plin2) were increased. Furthermore, correlation analysis across various diet-induced MASLD models revealed a strong positive association between TNFAIP3 and the expression of these lipogenesis-, inflammation-, and lipid regulation-related genes, as depicted in Fig. 1E. Importantly, TNFAIP3 levels were significantly elevated in the livers of patients with hepatic steatosis (Fig. 1F). Taken together, these findings strongly implicate that TNFAIP3 plays a critical role in regulating hepatic lipid metabolism and liver injury. However, the specific molecular mechanisms underlying this regulatory role of TNFAIP3 remain to be elucidated.

### TNFAIP3 protects against lipid accumulation in hepatocytes

**Fig. 2:**
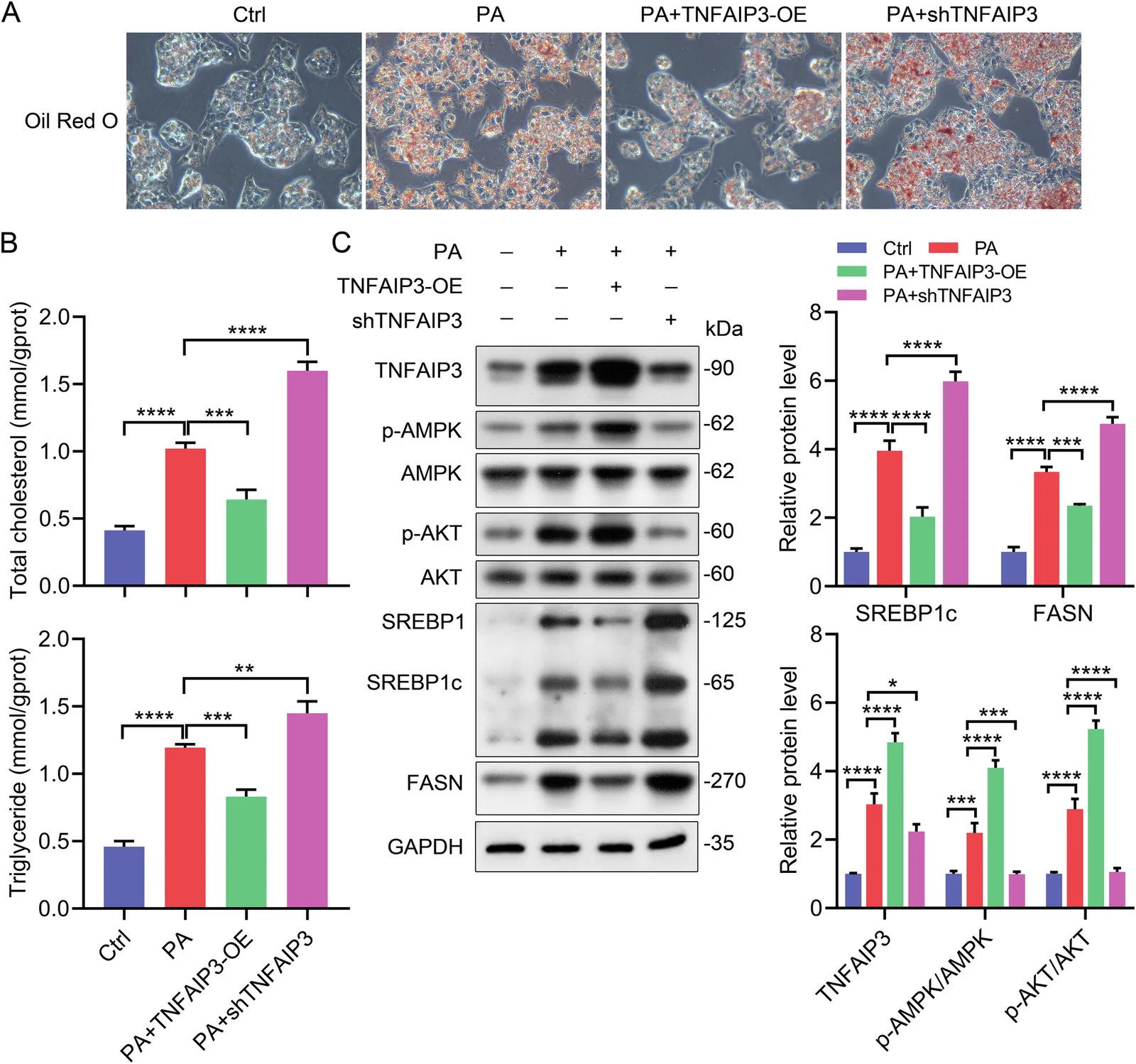
TNFAIP3 protects hepatocytes against lipid accumulation. **A** Representative Oil Red O staining of PA-treated HepG2 cells with TNFAIP3 overexpression or knockdown. **B** Intracellular levels of TC and TG following TNFAIP3 overexpression or knockdown. **C** Western blot analysis of TNFAIP3, p-AMPK, AMPK, p-AKT, AKT, SREBP1c and FASN in the indicated groups. *P < 0.05; **P < 0.01; ***P < 0.001; ****P < 0.0001.

To further investigate the role of TNFAIP3 in MASLD, Oil Red O staining was performed on PA-treated HepG2 cells. The results demonstrated that overexpression of TNFAIP3 significantly mitigated lipid droplet accumulation, whereas knockdown of TNFAIP3 remarkedly aggravated intracellular lipid deposition in vitro (Fig. 2A, Supplementary Fig. S2A, B). Consistent with these observations, TC and TG levels were reduced following TNFAIP3 overexpression and elevated upon TNFAIP3 silencing (Fig. 2B).

Phosphorylated AMP-activated kinase (AMPK) functions as an inhibitor of lipogenesis and cholesterol synthesis [13]. Additionally, phosphorylated protein kinase B (AKT) can further phosphorylate and inactivate pro-apoptotic proteins, thereby enhancing cell survival [14]. Mechanistically, TNFAIP3 overexpression resulted in downregulation of lipogenic markers sterol regulatory element-binding protein 1c (SREBP1c) and FASN, accompanied by enhanced phosphorylation of AMPK and AKT. These results suggest reduced lipogenesis and improved cellular metabolic homeostasis (Fig. 2C). Conversely, TNFAIP3 knockdown upregulated the expression of SREBP1c and FASN while decreasing the phosphorylation levels of AMPK and AKT, indicating increased lipogenesis and disrupted lipid homeostasis (Fig. 2C).

### TNFAIP3 alleviates PA-induced hepatic lipid accumulation via autophagy

**Fig. 3:**
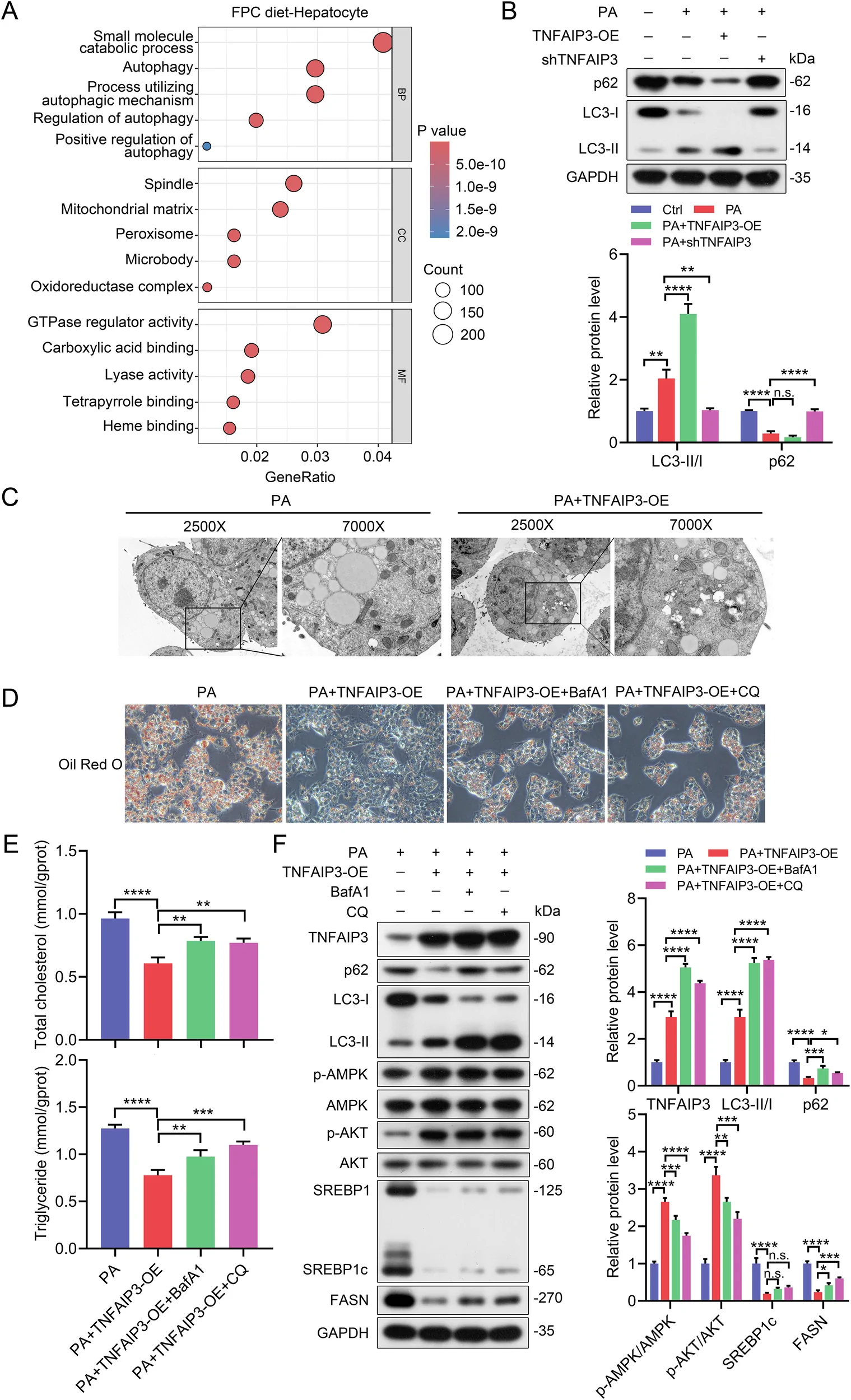
TNFAIP3 alleviates PA-induced hepatic lipid accumulation via autophagy. **A** GO enrichment analysis of differentially expressed genes between the high and low TNFAIP3 expression groups in hepatocytes from FPC diet-induced MASLD mouse models. **B** Western blot analysis of LC3-II and p62 protein levels in PA-treated HepG2 cells following TNFAIP3 overexpression or knockdown. **C** Transmission electron microscopy images of PA-treated HepG2 cells with TNFAIP3 overexpression. Representative Oil Red O staining (**D**) and intracellular levels of TC and TG (**E**) in PA-treated HepG2 cells overexpressing TNFAIP3, with or without co-treatment with the autophagy inhibitors BafA1 or CQ. **F** Western blot analysis of TNFAIP3, p62, LC3, p-AMPK, AMPK, p-AKT, AKT, SREBP1c and FASN in TNFAIP3-overexpressing HepG2 cells treated with or without BafA1 or CQ. *P < 0.05; **P < 0.01; ***P < 0.001; ****P < 0.0001; n.s. non-significant.

To elucidate the mechanisms by which TNFAIP3 regulates lipid metabolism, we stratified liver tissues or hepatocytes from various diet-induced MASLD mouse models into groups with high and low TNFAIP3 expression. Subsequent GO enrichment analysis revealed that differentially expressed genes in both hepatocytes and liver tissues were significantly enriched in autophagy-related biological processes (Fig. 3A, Supplementary Fig. S3A–C). Moreover, TNFAIP3 overexpression significantly increased LC3-II levels and decreased p62 expression, indicating enhanced autophagic activity. In contrast, TNFAIP3 knockdown reduced LC3-II levels and increased p62 levels, suggesting impaired autophagy in vitro (Fig. 3B). The promotion of autophagosome formation in hepatocytes by TNFAIP3 overexpression was further verified using transmission electron microscopy (Fig. 3C).

Notably, the TNFAIP3-mediated reduction in lipid droplet accumulation, as well as in TC, and TG levels, was reversed by treatment with the autophagy inhibitors BafA1 and CQ (Fig. 3D, E, Supplementary Fig S3D). Consistently, the inhibitory effects of TNFAIP3 on lipogenic gene expression were abolished by co-treatment with BafA1 or CQ, indicating that the regulatory role of TNFAIP3 in lipid metabolism is primary autophagy-dependent (Fig. 3F).

### TNFAIP3 alleviate hepatic steatosis and metabolic dysregulation via autophagy in vivo

**Fig. 4:**
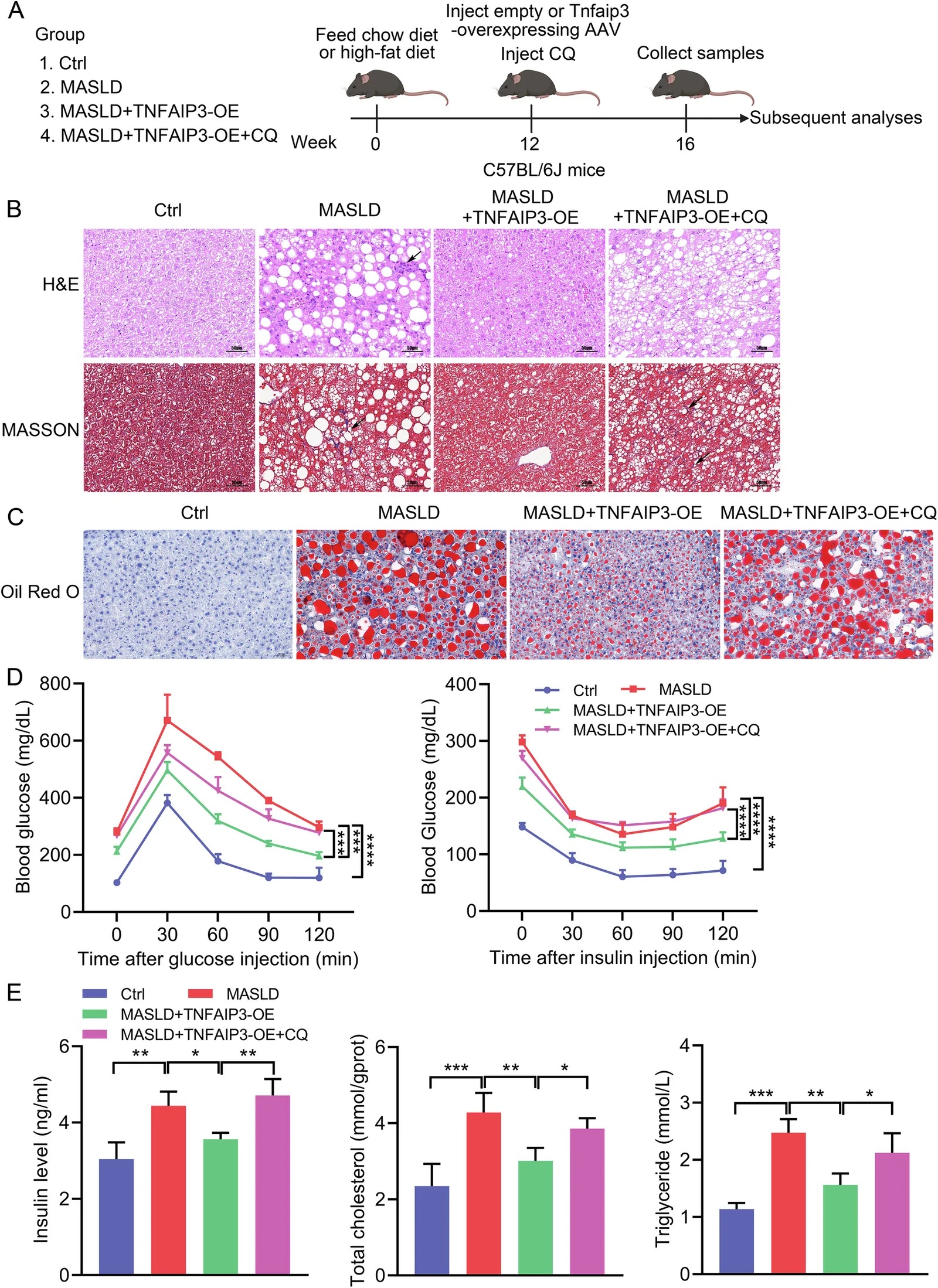
TNFAIP3 alleviates hepatic steatosis and improves metabolic homeostasis through autophagy in vivo. **A** Schematic diagram of the mouse model design. Representative H&E staining, Masson staining (**B**) and Oil Red O staining (**C**) of liver sections from HF diet-fed mice with TNFAIP3 overexpression, with or without CQ treatment. **D** GTT (left panel) and ITT (right panel) results in the indicated mouse groups. **E** Serum levels of insulin, TC and TG in the indicated groups. *P < 0.05; **P < 0.01; ***P < 0.001; ****P < 0.0001.

To validate the protective role of TNFAIP3 in MASLD in vivo, we established a TNFAIP3-overexpressing HF diet-induced mouse model, with or without administration of the autophagy inhibitor CQ (Fig. 4A). Consistent with our expectations, histopathological staining demonstrated that mice fed a HF diet exhibited markedly augmented lipid droplet accumulation, aggravated inflammatory cell infiltration, and substantial collagen deposition, compared with controls. Conversely, TNFAIP3 overexpression effectively rescued all these HF-induced pathological lesions (Fig. 4B, C, Supplementary Fig. S4A–C). TNFAIP3 overexpression also significantly improved fasting glucose levels, systemic glucose tolerance, and insulin sensitivity (Fig. 4D, Supplementary Fig S5). Consistently, serum TC and triglyceride TG levels were reduced, while insulin sensitivity was improved in TNFAIP3-overexpressing mice (Fig. 4E).

**Fig. 5:**
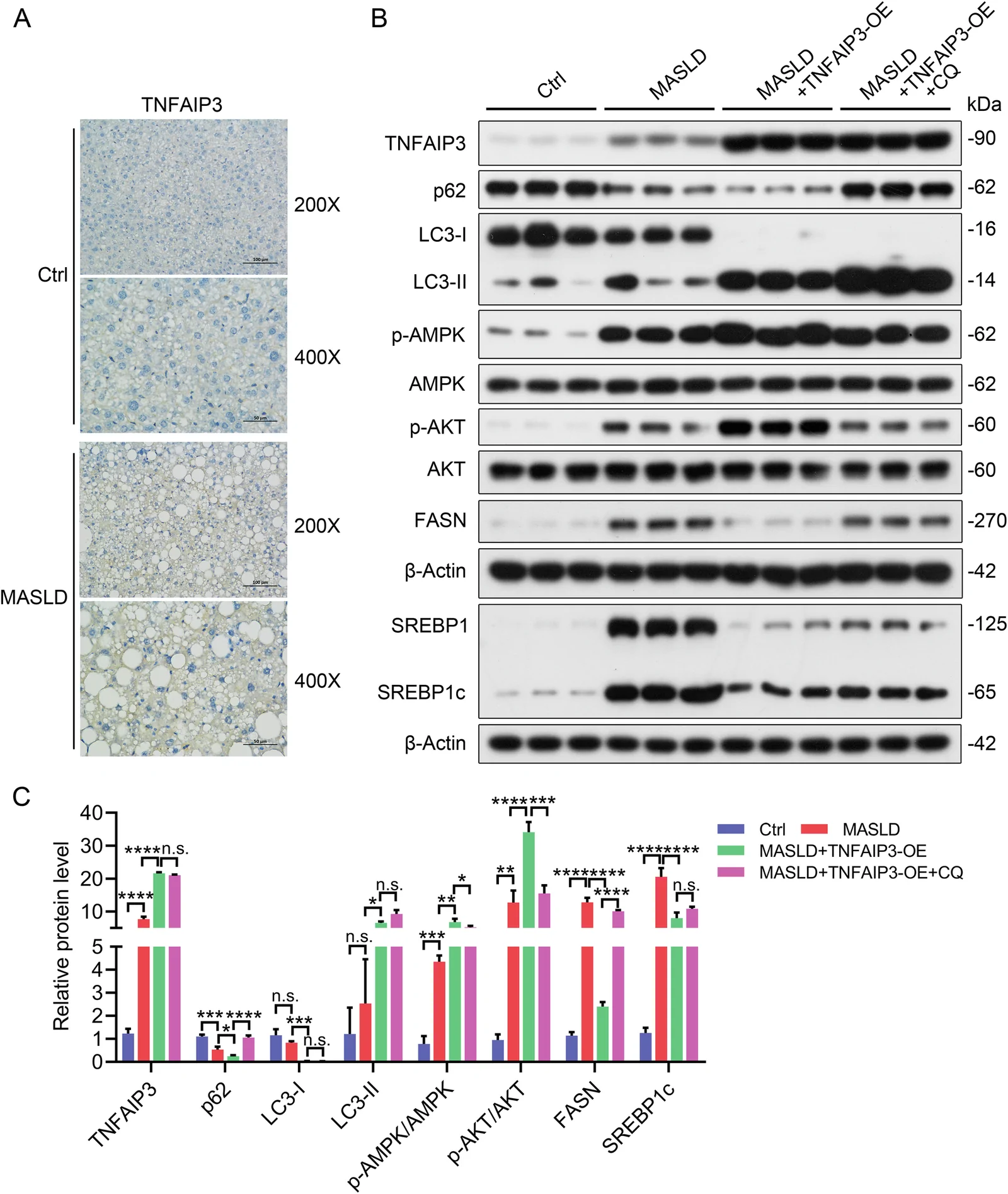
TNFAIP3 modulates lipid-associated metabolic pathways through an autophagy-dependent mechanism in vivo. **A** Representative immunohistochemical staining of hepatic TNFAIP3 in mice fed a HF diet. Western blot analysis (**B**) and semi-quantitative analysis (**C**) of TNFAIP3, p62, LC3, p-AMPK, AMPK, p-AKT, AKT, SREBP1c, and FASN in liver tissues. *P < 0.05; **P < 0.01; ***P < 0.001; ****P < 0.0001; n.s. non-significant.

Consistent with the above observations, immunohistochemical staining revealed that a HF diet induced upregulation of TNFAIP3 expression in hepatocyte cytoplasm (Fig. 5A, Supplementary Fig. S4D). Mechanistically, TNFAIP3 overexpression suppressed the expression of lipogenic genes SREBP1c and FASN, and activated the AMPK and AKT signaling pathways, as indicated by elevated phosphorylation levels (Fig. 5B, C). Enhanced autophagy was also confirmed by the upregulation of LC3-II and downregulation of p62. Notably, these effects were largely abolished by co-treatment with CQ, further supporting that TNFAIP3 exerts its metabolic benefits through an autophagy-dependent mechanism (Figs. 4B–E and 5B, C).

### TNFAIP3 promotes hepatocyte autophagy by stabilizing ATG4B via its deubiquitinating activity

**Fig. 6:**
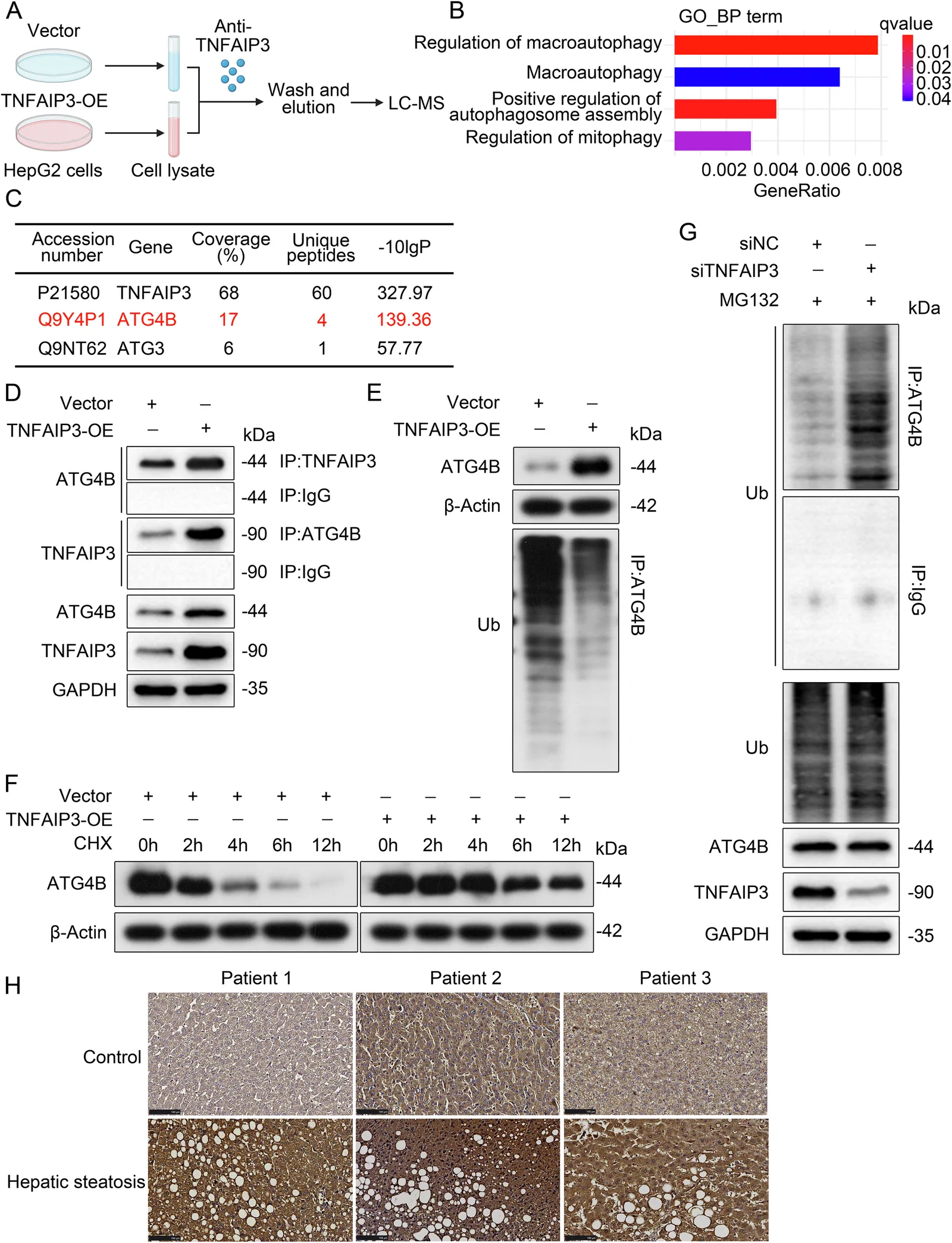
TNFAIP3 promotes hepatocyte autophagy by stabilizing ATG4B via its deubiquitinating activity. **A** Schematic workflow of mass spectrometry analysis for identifying TNFAIP3-interacting proteins. **B** GO enrichment analysis of TNFAIP3-bound proteins, highlighting significant enrichment in autophagy-related pathways. **C** Descriptions and scores of autophagy-related proteins differentially associated with TNFAIP3, identified by mass spectrometry. **D** Co-IP assays of TNFAIP3 and ATG4B followed by reciprocal Western blot analysis in TNFAIP3-overexpressing HepG2 cells. **E** Co-IP assays of ATG4B followed by Western blot analysis of ubiquitin in TNFAIP3-overexpressing HepG2 cells. **F** Western blot analysis of ATG4B at the indicated time points following CHX treatment in HepG2 cells with or without TNFAIP3 overexpression. **G** Western blot analysis of ATG4B and its ubiquitination in MG132-treated HepG2 cells transfected with TNFAIP3 siRNA (siTNFAIP3) or negative control siRNA (siNC). **H** Representative immunohistochemical staining of hepatic ATG4B in human livers.

To dissect the molecular mechanisms by which TNFAIP3 mediates autophagy, we performed immunoprecipitation with mass spectrometry to screen for TNFAIP3-interacting proteins (Fig. 6A, Supplementary Table S4). We identified 1183 proteins in the TNFAIP3 complex from the vector-treated control group, while 3072 interacting proteins were detected in TNFAIP3-overexpressing HepG2 cells. Among these, 1041 proteins overlapped between the vector and TNFAIP3-OE groups, suggesting that TNFAIP3 overexpression induced a substantial shift in the TNFAIP3-binding protein profile compared with the control group. Compared with controls, proteins bound to TNFAIP3 upon its overexpression were significantly enriched in autophagy-related pathways (Fig. 6B). Notably, among autophagy-related proteins [15], ATG4B, a core component of the autophagosome formation in mammalian cells, was identified as a key differentially associated protein (Fig. 6C). To further validate the physical association between TNFAIP3 and ATG4B, reciprocal Co-IP experiments were carried out. In cells overexpressing TNFAIP3, ATG4B was detectable within TNFAIP3 immunoprecipitates. Reciprocally, TNFAIP3 was enriched in ATG4B pull-down products relative to IgG negative controls (Fig. 6D). These results confirm that TNFAIP3 and ATG4B form a complex intracellularly. We then sought to determine whether the TNFAIP3-ATG4B protein complex governs autophagy activity. Relative to control cells, TNFAIP3 overexpression elevated the counts of yellow puncta (GFP+/mRFP+, autophagosomes), red puncta (GFP−/mRFP+, autolysosomes), alongside a higher autolysosome-to-autophagosome ratio, indicative of accelerated autophagic flux. Conversely, ATG4B silencing blunted this pro-autophagic phenotype triggered by TNFAIP3 overexpression (Supplementary Fig. S6).

Interestingly, GO enrichment analysis revealed a significant overrepresentation of proteasome-related biological processes among the top 20 enriched terms (Supplementary Fig. S7), suggesting a potential link between TNFAIP3 and proteasome-mediated protein degradation. Co-IP assays further demonstrated that TNFAIP3 overexpression markedly increased ATG4B protein levels and reduced its ubiquitination (Fig. 6E). Prediction using the GPS-Uber database indicated that ATG4B harbors multiple putative ubiquitination sites (Supplementary Table S5). Following cycloheximide (CHX) treatment, Western blot analysis showed that ATG4B degradation was significantly delayed in TNFAIP3-overexpressing HepG2 cells, indicating that TNFAIP3 stabilizes ATG4B by inhibiting ubiquitin-mediated proteasomal degradation, potentially via its deubiquitinating activity, thereby promoting autophagy (Fig. 6F). Consistently, TNFAIP3 depletion markedly enhanced ATG4B ubiquitination in HepG2 cells (Fig. 6G). Notably, pharmacological inhibition of the proteasome with MG132 fully rescued the reduction in total ATG4B protein abundance triggered by TNFAIP3 silencing, indicating that ATG4B degradation is mediated through the ubiquitin-proteasome system. Moreover, ATG4B expression was also upregulated in the liver tissues of patients with hepatic steatosis, a pattern consistent with that of TNFAIP3 (Fig. 6H).

**Fig. 7:**
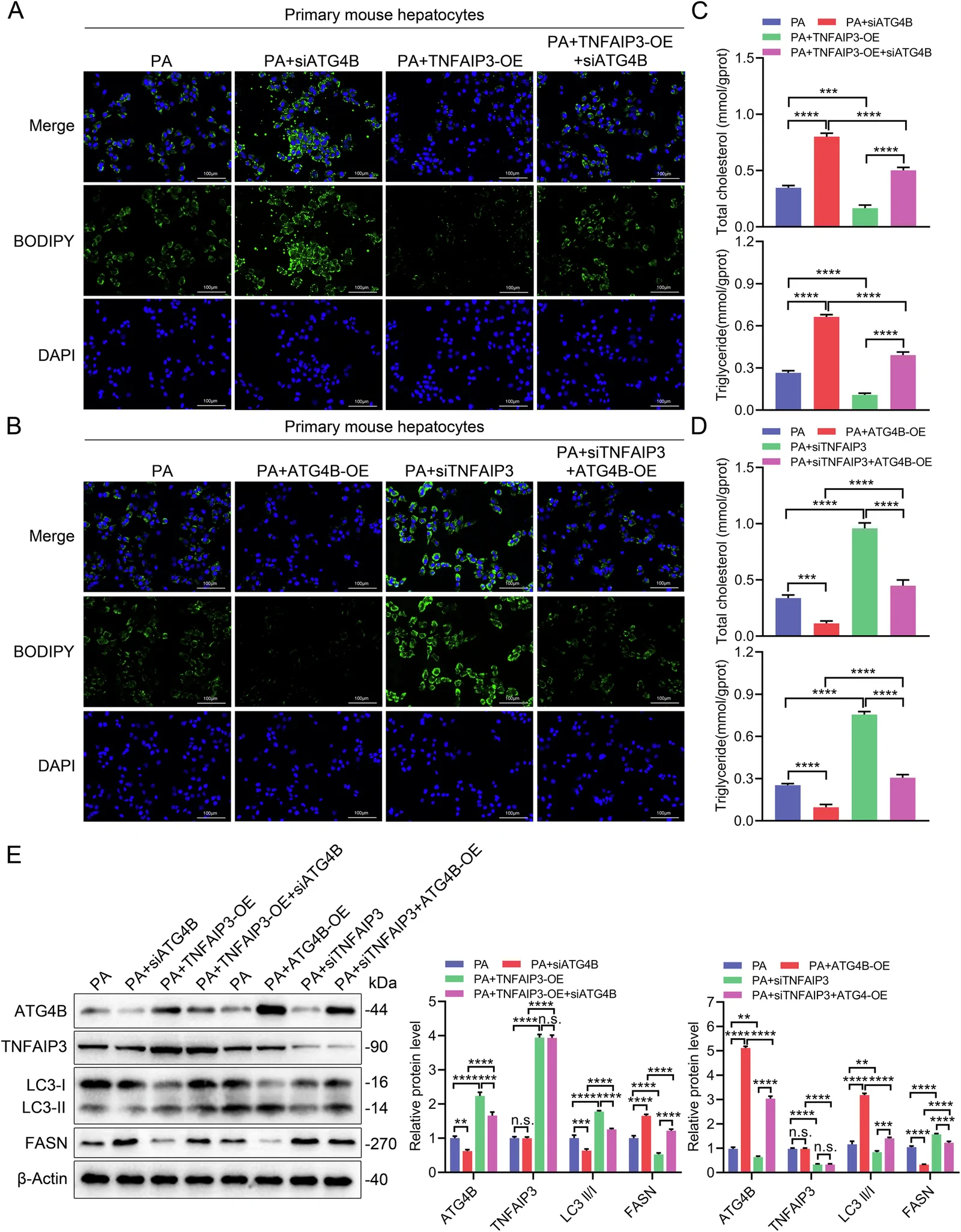
TNFAIP3-ATG4B axis modulates hepatic lipid accumulation. Representative immunofluorescence images of lipid droplets (**A**) and intracellular TC and TG levels (**C**) in PA-treated primary mouse hepatocytes subjected to ATG4B knockdown, TNFAIP3 overexpression, or combined TNFAIP3 overexpression and ATG4B knockdown. Representative immunofluorescence images of lipid droplets (**B**) and intracellular TC and TG levels (**D**) in PA-treated primary mouse hepatocytes subjected to ATG4B overexpression, TNFAIP3 knockdown, or combined TNFAIP3 knockdown and ATG4B overexpression. **E** Western blot analysis of TNFAIP3, ATG4B, LC3, and FASN expression in PA-treated primary mouse hepatocytes following manipulation of TNFAIP3 and ATG4B expression. **P < 0.01; ***P < 0.001; ****P < 0.0001; n.s. non-significant.

To further investigate whether TNFAIP3 regulates hepatic steatosis via an ATG4B-dependent mechanism, we manipulated the expression of ATG4B and TNFAIP3 in primary murine hepatocytes (Fig. 7) and HepG2 cells (Supplementary Fig. S9). Specifically, we implemented combinatorial interventions involving ATG4B knockdown plus TNFAIP3 overexpression, and ATG4B overexpression plus TNFAIP3 knockdown, followed by assessment of intracellular lipid droplet accumulation. We found that either knockdown of ATG4B or TNFAIP3 resulted in increased lipid droplet accumulation, whereas either overexpression of ATG4B or TNFAIP3 attenuated this phenotype (Supplementary Fig. S8, Fig. 7A, B, Supplementary Fig. S9A, B). Notably, ATG4B overexpression alleviated lipid accumulation in the context of TNFAIP3 knockdown, while ATG4B knockdown exacerbated lipid accumulation following TNFAIP3 overexpression (Fig. 7A, B, Supplementary Fig. S9A, B). Quantitative measurements of TC and TG levels (Fig. 7C, D, Supplementary Fig. S9C, D), as well as protein expression of FASN (Fig. 7E, Supplementary Fig. S9E), corroborated the results of lipid droplet staining. As anticipated, knockdown of ATG4B and TNFAIP3 reduces the LC3II/I ratio, while their overexpression increases this autophagy-associated index. Overexpressing ATG4B in TNFAIP3-knockdown cells restores LC3II/I ratio, whereas ATG4B silencing in TNFAIP3-overexpressing cells diminished this ratio (Fig. 7E, Supplementary Fig. S9E). Collectively, these findings indicated that TNFAIP3 and ATG4B function in concert to regulate autophagy.

### NF-κB signaling pathway drives TNFAIP3 upregulation in MASLD

**Fig. 8:**
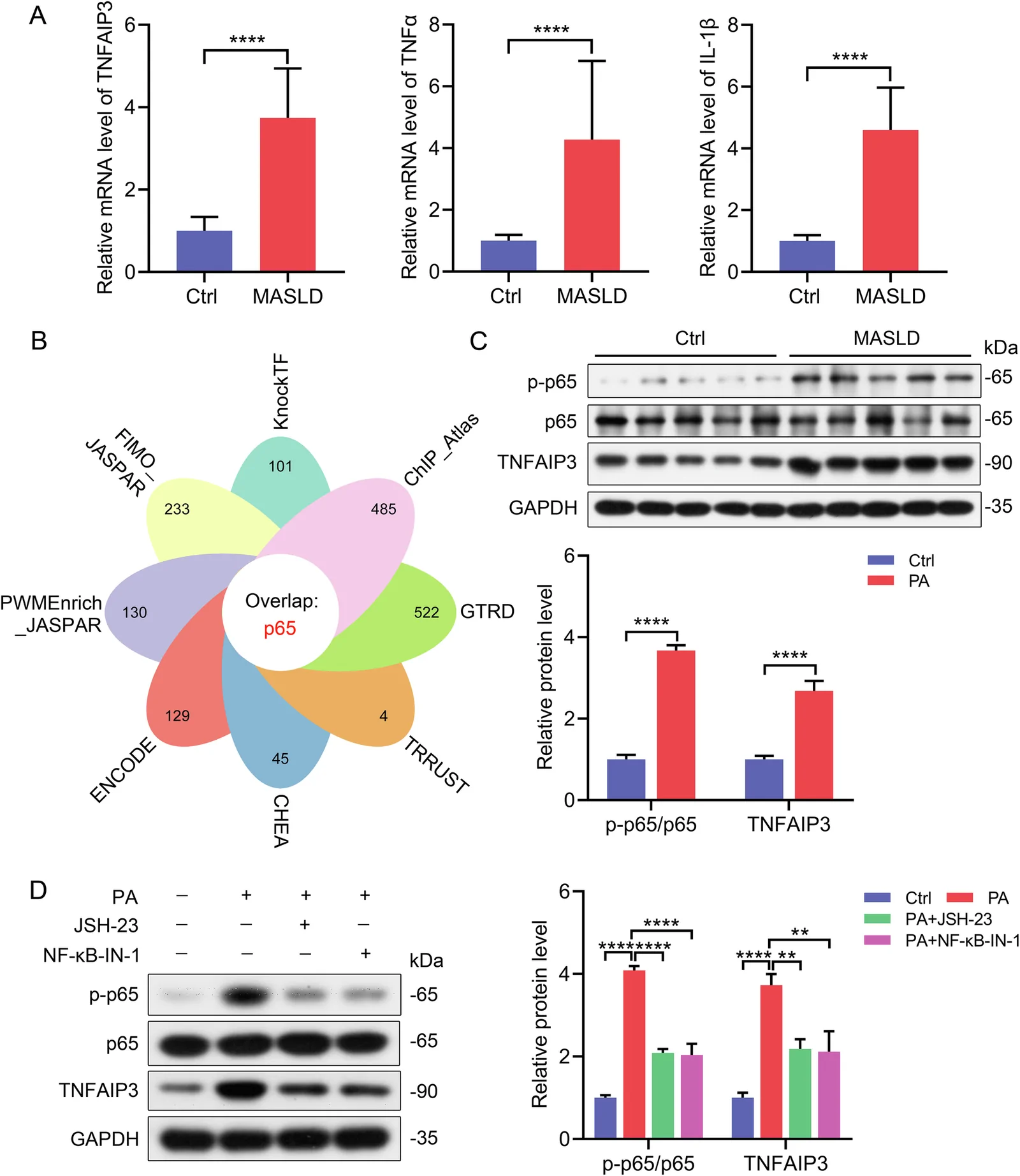
NF-κB signaling pathway drives TNFAIP3 upregulation in MASLD. **A** RT-qPCR analysis of hepatic TNFAIP3, TNFα, and IL1β mRNA levels in HF diet-fed mice. **B** Bioinformatic prediction of TNFAIP3 transcription factors based on multiple databases. **C** Western blot analysis of hepatic p-NF-κB and TNFAIP3 expression in HF diet-fed mice. **D** Western blot analysis of p-NF-κB and TNFAIP3 protein levels following treatment with NF-κB inhibitors JSH-23 or NF-κB-IN-1 in PA-treated HepG2 cells. **P < 0.01; ****P < 0.0001.

To investigate the upstream regulatory mechanisms of TNFAIP3 in MASLD, we first examined whether TNFAIP3 is transcriptionally regulated. RT-qPCR analysis showed that the mRNA levels of TNFAIP3, as well as those of the pro-inflammatory cytokines TNFα and IL1β, were significantly elevated in HF diet-fed mice (Fig. 8A). To further identify potential transcription factors of TNFAIP3, we performed in silico analyses using multiple databases, including KnockTF, ChIP-Atlas, GTRD, TRRUST, CHEA, ENCODE, FIMO_JASPAR, and PWMEnrich_JASPAR. Intriguingly, v-rel reticuloendotheliosis viral oncogene homolog A (RELA, also referred to as p65), a key subunit of the NF-κB complex, was consistently predicted as a major transcriptional regulator of TNFAIP3 (Fig. 8B). Western blot and RT-qPCR analyses further confirmed that both p-p65 and TNFAIP3 levels were upregulated in HF diet-fed mice (Fig. 8C). Importantly, pharmacological inhibition of NF-κB activation using JSH-23 or NF-κB-IN-1 significantly decreased the protein levels of p-p65 and TNFAIP3, indicating that TNFAIP3 upregulation in MASLD is, at least in part, mediated by the NF-κB signaling pathway (Fig. 8D). Consistently, dual-luciferase reporter assays further demonstrated that PA significantly stimulated TNFAIP3 promoter activity in HepG2 cells, while pharmacological suppression of NF-κB substantially blunted this induction (Supplementary Fig. S10).

## Discussion

Hepatic autophagy is a crucial cellular process that not only degrades cytoplasmic components but also maintains metabolic homeostasis through quality control, lipophagy, and regulation of signaling molecules [16, 17].

In preclinical models, enhanced autophagy has been demonstrated to alleviate hepatic steatosis and liver damage in metabolic liver disorders [18, 19]. Interestingly, liver-specific Atg5 knockout mice exhibited reduced hepatic triglyceride content, suggesting that autophagy is required not only for lipid droplet degradation but also for lipid biogenesis [20, 21]. A recent study reported that liver-specific overexpression of ATG9A, acting as a dual metabolic effector, enhances autophagic flux while impairing autophagosome degradation, thereby disrupting hepatic lipid metabolism and exacerbating inflammation and fibrosis [22]. In addition, liver-specific deletion of Atg7 protected mice from diet-induced obesity and improved insulin resistance through mitochondrial dysfunction-mediated fibroblast growth factor 21 signaling in autophagy-deficient livers [23]. Similarly, hepatocyte-specific deletion of focal adhesion kinase family-interacting protein of 200 kDa prevented diet- or starvation-induced hepatic fat accumulation and downregulated lipid metabolism genes [24]. Furthermore, hepatic autophagy was stimulated upon exposure to lipopolysaccharide, while its absence rendered mice more susceptible to endotoxin-induced liver injury [24]. These findings indicate that while autophagy deficiency alone does not necessarily exacerbate hepatic steatosis, autophagy plays a protective role against gut-derived endotoxins, and its inhibition may accelerate the progression of MASH [24]. Taken together, the role of autophagy in MASLD remains incompletely understood, and therapeutic modulation of autophagy may represent a promising strategy for the treatment of MASLD.TNFAIP3 acts as an essential factor in liver protection through multiple hepatic cell types and signaling pathways. TNFAIP3-mediated inhibition of NF-κB signaling in macrophages has been shown to protect against insulin resistance, glucose dysregulation, and hepatic steatosis induced by a HF diet [25]. Additionally, TNFAIP3 regulates chemokine expression in hepatic stellate cells, delaying the progression of liver inflammation [26]. In hepatocyte-specific TNFAIP3 knockout mice, chronic liver inflammation and increased hepatocyte apoptosis were associated with an enhanced susceptibility to hepatocellular carcinoma development following HF diet-induced stress [27]. Moreover, partial hepatectomy led to an unexpectedly high mortality rate in TNFAIP3 heterozygous mice due to aggravated steatosis, highlighting the vital role of TNFAIP3 in hepatocyte proliferation [28].

Herein, TNFAIP3 expression was significantly increased in steatotic human livers, diet-induced mouse livers and isolated primary hepatocytes, suggesting that this elevation occurs specifically in parenchymal liver cells, rather than being restricted to non-parenchymal compartments. Furthermore, a strong positive correlation between TNFAIP3 and the expression of genes involved in lipogenesis, inflammation, and lipid regulation across different diet-induced MASLD models indicated the upregulation of TNFAIP3 as integral to the anti-inflammatory and metabolic regulatory mechanisms in hepatocytes. As demonstrated in MASLD cell and mouse model, overexpression of TNFAIP3 alleviated lipid accumulation and improved cellular metabolic homeostasis by downregulating lipogenic markers and promoting AMPK/AKT phosphorylation, whereas TNFAIP3 knockdown exerted the opposite effect.

As previously reported, TNFAIP3 promotes autophagy through MAP1LC3/LC3 puncta formation in CD4^+^ T cells [29]. However, TNFAIP3 also acts as a negative regulator of oxidative stress-induced autophagy by inhibiting TNF receptor-associated factor 6-mediated ubiquitination of ATG9A [30]. Thus, the role of TNFAIP3 in autophagy remains controversial across different disease contexts, and its involvement in autophagy in MASLD has yet to be elucidated. In this study, TNFAIP3 enhanced autophagic activity; notably, the inhibitory effects of TNFAIP3 on lipids accumulation and lipogenic gene expression were abolished by co-treatment with autophagy inhibitors both in vitro and in vivo, suggesting that the regulatory role of TNFAIP3 in lipid metabolism is primarily autophagy-dependent. Additionally, a previous study demonstrated that HF diet-induced inhibition of autophagic flux reduces cryptochrome 1 protein levels in an autophagy-dependent manner, thereby exacerbating obesity-related hyperglycemia [16]. Consistent with this finding, we observed that HF diet significantly impaired insulin sensitivity and glucose regulation, whereas TNFAIP3 overexpression ameliorated these abnormalities and reduced insulin levels. However, this improvement was abrogated following treatment with the autophagy inhibitor. It has also been reported that TNFAIP3 promotes autophagy to protect certain cell types from death [4]. In the current study, TNFAIP3 appeared to promote cell survival by increasing p-AKT levels.

As a dual-ubiquitin-editing enzyme, the N-terminal OTU domain of TNFAIP3 mediates deubiquitination activity. It has been reported that TNFAIP3 inactivated the hyperactivated form of apoptosis signal-regulating kinase 1 by specifically removing polyubiquitination from the kinase [5]. Additionally, TNFAIP3 is recruited to and disrupts the K63-linked polyubiquitination of TNF receptor-associated factor 6 in hepatocytes [31]. In the present study, we identified that TNFAIP3 reduced the ubiquitination of ATG4B, thereby decreasing its proteasomal degradation and promoting autophagy.

Of note, the transcriptional levels of TNFAIP3 and classic downstream factors of NF-κB, IL-1β and TNFα, were significantly upregulated in MASLD. Furthermore, through publicly available data and using NF-κB inhibitors, this study confirmed that the NF-κB subunit p65 was an upstream transcription factor of TNFAIP3. It is well established that TNFAIP3 negatively regulates NF-κB activation through its dual-ubiquitin-editing function on NF-κB signaling intermediates and substrates in response to proinflammatory stimuli [4, 13, 32]. Collectively, NF-κB and TNFAIP3 are involved in a delicate feedback loop that orchestrates liver protection.

Interestingly, a recent study has revealed that NF-κB activation in hepatocytes induces hepatic steatosis without triggering inflammation, and the loss of hepatocyte TNFAIP3 further exacerbates steatosis [13]. Furthermore, deregulated NF-κB activation exerts a significant impact on the development of hepatic steatosis, insulin resistance, inflammation, fibrosis, and cancer [33]. Indeed, the effects of NF-κB are complex and multifactorial. The seemingly contradictory outcomes may stem from the pleiotropic actions of NF-κB in different cell types, each of which plays distinct and often divergent roles during liver injury [33]. Therefore, it remains to be further investigated whether the NF-κB-TNFAIP3 feedback loop plays distinct roles in different hepatic cell types (hepatocytes and non-parenchymal cells), tissues (adipose tissue, intestine, liver), or in the context of liver inflammation and steatosis, thereby directly or indirectly influencing the progression of MASLD.

This present study still has several limitations. First, the relatively small human clinical cohort necessitates cautious interpretation of our findings. We were unable to conduct stratified analysis of hepatic steatosis patient samples according to TNFAIP3 and ATG4B expression levels, owing to insufficient sample quantity and absence of supporting transcriptomic data. In addition, we did not fully dissect the precise molecular mechanism by which TNFAIP3 governs ATG4B ubiquitination in the present study. We plan to resolve these drawbacks in subsequent research, which will encompass expanded clinical cohorts paired with transcriptomic profiling, alongside mechanistic assays utilizing TNFAIP3 OTU domain mutants and ubiquitin linkage-specific mutants.

In summary, this study demonstrates that TNFAIP3, which is upregulated by the NF-κB signaling pathway, plays a protective role in the progression of MASLD by reducing the ubiquitination and proteasomal degradation of ATG4B and enhancing autophagy. Thus, the NF-κB-TNFAIP3 feedback loop in hepatocytes may represent a potential therapeutic target for MASLD.

## Materials and methods

### Plasmid construction

The expression plasmids p-TNFAIP3 and p-ATG4B were generated by cloning the full-length coding sequences of TNFAIP3 and ATG4B, respectively, into the pcDNA3.1 expression vector (Asia-Vector Biotechnology, China). Gene-specific primers (h-TNFAIP3-F: 5’-CTCGGATCCGCCACCATGGCTGAACAAGTCCTTCC-3’; h-TNFAIP3-R: 5’-CCCTCTAGACTCGAGGCCATACATCTGCTTGAACT-3’; h-ATG4B-F: 5’-CTCGGATCCGCCACCATGGACGCAGCTACTCTGAC-3’; h-ATG4B-R: 5’-CCCTCTAGACTCGAGAAGGGACAGGATTTCAAAGT-3’) were utilized for PCR amplification of the corresponding coding regions. The pcDNA3.1 vector was linearized via digestion with restriction enzymes XhoI and BamHI (NEB, USA) and subsequently subjected to purification. Exonuclease III (BioFriend, China) -mediated ligation-independent cloning was performed to assemble the PCR-amplified fragments with the linearized vector. The resulting ligation products were transformed into E. coli DH5α competent cells, and positive recombinant clones were validated via colony PCR followed by Sanger sequencing.

### Cell transfection and treatment

The human hepatoma cell line HepG2 (American Type Culture Collection, USA) was cultured in Dulbecco’s Modified Eagle’s Medium (DMEM, BasalMedia, China) supplemented with 10% fetal bovine serum (NEWZERUM, New Zealand), 100IU/mL penicillin, and 100 μg/mL streptomycin (Sangon Biotech, China). Then HepG2 cells were seeded onto plates pre-coated with rat tail Collagen I.

Primary mouse hepatocytes were cultured in high-glucose DMEM supplemented with 10% FBS and 1% penicillin/streptomycin. The culture medium was further supplemented with insulin (10 nM), dexamethasone (50 nM), EGF (10 ng/mL), HGF (5 ng/mL), and GSH (0.1–1 mM). Cells were seeded and maintained on Matrigel-precoated culture plates.

Subsequently, the cells were transfected with the indicated dose of plasmids, shRNA or siRNA, respectively. At forty-eight hours post-transfection, both cell supernatants and the whole-cell lysates were harvested for subsequent downstream assays.

For the construction of the MASLD cell model, after transfection with plasmids or shRNA, HepG2 cells were treated with 300 μM PA for 24 h. To investigate the role of autophagy in this process, either Bafilomycin A1 (BafA1) (0.1 μM, MedChemExpress, MCE, USA) or chloroquine (CQ) (10 μM, MCE, USA) was added concurrently with the PA treatment. And after 24 h of this combined intervention, the cells were collected for further molecular and biochemical analysis. To investigate the mechanism by which TNFAIP3 regulates ATG4B, 10 μM cycloheximide (CHX) was added to the culture medium of HepG2 cells to inhibit protein synthesis. To investigate whether TNFAIP3 inhibits proteasomal degradation of ATG4B via deubiquitination, HepG2 cells were treated with MG132 for 4–6 h before harvest. In additional experiments, specific inhibitors were used to explore relevant signaling pathways. NF-κB inhibitors JSH-23 (MCE, USA) was applied at a concentration of 25 μM for 4 h, while NF-kB-IN-1 (MCE, USA) was administered at 5 μM for the same duration. The treated cells were then processed according to requirements for subsequent investigation.

### Human liver specimens and animal models

Human liver specimens were obtained from adjacent non-tumorous tissues of patients who underwent surgical resection for hepatocellular carcinoma. According to the degree of fatty infiltration in the adjacent liver tissue, the samples were divided into two groups: a control group with minimal or absent steatosis, and a hepatic steatosis group. The patients’ characteristics are summarized in Supplementary Table S1. The use of human liver specimens in this study was approved by the Ethics Committee of Shanghai Sixth People’s Hospital (Approval No. 2020-069). Written informed consent was obtained from all patients prior to sample collection.

Six-week-old male specific pathogen-free C57BL/6J mice were obtained from Beijing Vital River Laboratory Animal Technology Co., Ltd. (VRL, China). After a 2-week acclimatization period to the environment, the mice were randomly allocated into four experimental groups with five mice in each group: (1) Control group fed with regular chow diet; (2) MASLD group fed with a high-fat diet (D12492, VRL, China); (3) MASLD + TNFAIP3-OE group, in which mice received a HF diet and were intravenously injected via the tail vein with adeno-associated virus (AAV) vectors overexpressing TNFAIP3 at a dosage of 1 × 10¹² viral genomes per mouse; and (4) MASLD + TNFAIP3-OE + CQ treatment group, which received HF diet and the same AAV-TNFAIP3 intervention along with intraperitoneal administration of CQ at a daily dose of 50 mg/kg. The HF diet regimen lasted for 16 weeks, while the adenovirus and CQ interventions were initiated during the final 4 weeks of the feeding period.

The mice were housed in a temperature- and humidity-controlled facility with a strict 12-h light/dark cycle, and were allowed unrestricted access to food and water throughout the experiment. At the conclusion of the experimental period, after a 12 h overnight fast, the mice were euthanized. Serum and liver tissues were promptly collected and stored at −80 °C for subsequent analyses. All experimental procedures were performed in strict accordance with the guidelines approved by the Institutional Animal Care and Ethics Committee (Approval No.AD2022189).

### Biochemical analyses, glucose tolerance test (GTT) and insulin tolerance test (ITT)

Triglyceride (TG) and total cholesterol (TCHO) levels in both HepG2 cells and murine serum were quantified using commercial assay kits (Asia-Vector Biotechnology, China), according to the manufacturer’s instructions. Serum insulin levels were determined by enzyme-linked immunosorbent assay. For GTT, mice were fasted for 12 h followed by intraperitoneally injection of glucose (2 g/kg body weight). For ITT, mice were fasted for 12 h prior to intraperitoneal injection of insulin (0.75U/kg body weight). Blood glucose levels were measured via tail snip at 0, 30, 60, 90, and 120 min post-injection to assess glucose homeostasis and insulin sensitivity.

### Hematoxylin & eosin (H&E), Masson, Oil Red O, immunofluorescence and immunohistochemical staining

To evaluate hepatic damage and fibrosis, mouse liver tissues were fixed in 4% paraformaldehyde for 24 h and then embedded in paraffin. Paraffin-embedded sections were subjected to H&E and Masson staining.

To assess lipid accumulation, frozen liver sections and cultured cells were fixed (tissue sections with a general fixative; cells with 4% paraformaldehyde), rinsed, and equilibrated in 60% isopropanol. Samples were stained with freshly prepared Oil Red O working solution (a 3:2 mixture of saturated Oil Red O in isopropanol and water) for 8–10 min, followed by brief differentiation in 60% isopropanol and rinsing with distilled water. Nuclei were counterstained with hematoxylin, and slides were mounted with glycerol gelatin for microscopic analysis.

Additionally, the immunofluorescence staining was performed to evaluate the extent of lipid deposition. Cells were rinsed with PBS, fixed, and then rinsed thoroughly again. BODIPY 493/503 staining solution was applied dropwise onto the cells, which were then incubated at room temperature for 30 min. After incubation, the cells were rinsed with PBS and counterstained with Hoechst 33342 or DAPI for 5 min. Following a final PBS rinse, lipid droplets were visualized under a fluorescence microscope.

To assess the expression levels of TNFAIP3 and ATG4B in murine and human liver tissues, paraffin-embedded liver tissue sections were subjected to immunohistochemical staining with respective antibodies against TNFAIP3 (Abcam, UK; Proteintech, China) or ATG4B (Proteintech, China).

### Reverse-transcription quantitative real-time PCR

Total RNA was extracted from HepG2 cells or mouse liver tissues using TRIzol reagent (Invitrogen, USA) according to the manufacturer’s instructions. cDNA was synthesized via reverse transcription using the TransScript® All-in-One First-Strand cDNA Synthesis SuperMix for qPCR (TransGen Biotech, China). Quantitative real-time PCR was conducted using the AceQ Universal SYBR qPCR Master Mix (Vazyme, China) on a QuantStudio 5 Real-Time PCR System (Applied Biosystems, ThermoFisher Scientific, USA). Gene expression levels were normalized to GAPDH, and relative expression levels were calculated using the 2^−ΔΔCt^ method. The primer sequences in this study are listed below: Human: h-GAPDH: Forward: GGAGCGAGATCCCTCCAAAAT, Reverse: GGCTGTTGTCATACTTCTCATGG; h-TNFAIP3: Forward: 5’-TCCTCAGGCTTTGTATTTGAGC-3’, Reverse: 5’-TGTGTATCGGTGCATGGTTTTA-3’; Mouse: m-GAPDH-F: 5’-TGGCCTTCCGTGTTCCTAC-3’, m-GAPDH-R: 5’-GAGTTGCTGTTGAAGTCGCA-3’; m-TNFAIP3-F: 5’-TCCTCAGGCTTTGTATTTGAGC-3’, m-TNFAIP3-R: 5’-TGTGTATCGGTGCATGGTTTTA-3’; m-IL1β-F: 5’-GAAATGCCACCTTTTGACAGTG-3’, m-IL1β-R: 5’-TGGATGCTCTCATCAGGACAG-3’; m-TNFα-F: 5’-GATCGGTCCCCAAAGGGATG-3’; m-TNFα-R: 5’-CCACTTGGTGGTTTGTGAGTG-3’.

### Western blot analysis

Total protein extraction from HepG2 cells or mouse liver tissues was performed by lysing the samples in RIPA buffer supplemented with protease and phosphatase inhibitors on ice. Subsequently, the lysates underwent sonication and were centrifugated at 13,000 rpm for 10 min at 4 °C. Protein concentration was quantified using the Bradford assay. Equal amounts of protein samples were denatured at 100 °C for 10 min in loading buffer, separated - by SDS-PAGE, and then transferred onto PVDF membranes. The membranes were blocked with 5% non-fat milk in TBST for 1 h at room temperature. After blocking, they were incubated with primary antibodies overnight at 4 °C, followed by incubation with HRP-conjugated secondary antibodies (Cell Signaling Technology, USA) at 37 °C for 1 h. Following thorough washing steps, protein signals were detected using enhanced chemiluminescence reagents and visualized by autoradiography. Details of the primary antibodies used are presented in Supplementary Table S2.

### Mass spectrometry analysis

To identify TNFAIP3-enriching proteins, samples subjected to TNFAIP3 immunoprecipitation were separated by SDS-PAGE, and the target protein bands were excised from the gel. The gel pieces were dried, reduced with dithiothreitol, alkylated with iodoacetamide, and digested overnight with trypsin. The resulting peptides were extracted with acetonitrile-formic acid solutions, pooled, and lyophilized for mass spectrometry analysis.

### Co-immunoprecipitation (Co-IP)

For TNFAIP3 or ATG4B immunoprecipitation, cell lysates were incubated with TNFAIP3 or ATG4B antibody at 4 °C overnight. The antibody-antigen complexes were then incubated with Protein A/G Magnetic Beads (Vazyme) overnight. After extensive washing, proteins were eluted with loading buffer and boiled for 5 min. Proteins were separated by SDS-PAGE at 80–100 V and transferred from the gel to a PVDF membrane. The membrane was blocked, incubated with primary antibody (Supplementary Table S2) overnight at 4 °C, followed by HRP-conjugated secondary antibody for 1 h at 37 °C. The signal was detected using enhanced chemiluminescence kit and visualized by exposure to X-ray film.

### Luciferase reporter assay

Cells were co-transfected with a firefly luciferase reporter plasmid carrying the TNFAIP3 promoter, alongside a constitutively active Renilla luciferase plasmid serving as an internal reference control. Post-transfection, cells were subjected to treatment with PA alone, or co-treatment of PA combined with either JSH-23 or NF-κB-IN-1, respectively. Cells were subsequently lysed, and dual-luciferase activity was sequentially quantified following the manufacturer’s standard protocols. Briefly, firefly luciferase luminescence was initially measured upon addition of LAR II substrate., Stop & Glo® Reagent was then added to detect Renilla luciferase activity. All luminescence readouts were acquired using a luminometer. Raw firefly luciferase signals were normalized to corresponding Renilla luciferase values, and relative TNFAIP3 promoter activity was determined as the firefly-to-Renilla luminescence ratio.

### Autophagic flux detection

HepG2 cells were transfected with the TNFAIP3 overexpression plasmid, siATG4B, and their respective empty vector or siRNA negative control, alongside the mRFP-GFP-LC3 adenovirus reporter. Forty-eight hours post-transfection, cells were fixed in 4% paraformaldehyde, and GFP (green) and mRFP (red) fluorescence signals were visualized under a laser scanning confocal microscope. In merged fluorescent images, yellow puncta (GFP+/mRFP+) corresponded to LC3-labeled autophagosomes, while red-only puncta (GFP−/mRFP+) indicated LC3 structures within acidic lysosomal compartments, namely autolysosomes. The abundance and distribution of yellow and red puncta were quantitatively analyzed to assess alterations in autophagosome biogenesis and autophagic maturation.

### Bioinformatics analysis

The datasets analyzed in this study include GSE256063, GSE246328, GSE256501, and GSE256502 from the public GEO database (Supplementary Table S3). Differentially expressed genes (DEGs) were identified using the DESeq2 package. Genes with an absolute log₂ fold change greater than 0.5 and an adjusted *P*-value less than 0.05 were considered statistically significant. Gene Ontology (GO) enrichment analysis of DEGs was performed using the ClusterProfiler package to identify significantly enriched biological processes. In addition, Spearman correlation analysis was conducted.

Prediction of potential ubiquitination sites on ATG4B was performed using the GPS-Uber database, which evaluates lysine residues for putative ubiquitination based on sequence motifs and scoring thresholds.

The transcription factor prediction was performed using multiple databases, including KnockTF, ChIP-Atlas, GTRD, TRRUST, CHEA, ENCODE, PWMEnrich_JASPAR, and FIMO_JASPAR.

### Statistical analyses

Numerical data are presented as mean ± SD. Data analysis was performed using GraphPad Prism 8 software. For comparisons between two groups, an unpaired *t*-test was employed. In the case of multiple groups, one-way analysis of variance (ANOVA) was used to assess statistical significance. To analyze changes in GTT and ITT over time, two-way ANOVA was applied. A *p*-value of less than 0.05 was considered as statistically significant.

## Supplementary information

Supplementary materials-The NF-κB-TNFAIP3 Axis Attenuates MASLD via Activation of Hepatocyte Autophagy

Western blot original files

## Data Availability

The RNA-seq datasets were downloaded from http:// www.ncbi.nlm.nih.gov/ with the accession numbers GSE256063, GSE246328, GSE256501, and GSE256502. Data related to this paper are available on reasonable request.
