# Supplementary material for: The NF-κB-TNFAIP3 axis attenuates MASLD via activation of hepatocyte autophagy
Source: Cell Death Discov. 2026 Aug 25;12:430. doi: 10.1038/s41420-026-03310-9 (PMC13720041; doi:10.1038/s41420-026-03310-9)

# Western blot original files

**Figure 2C:**

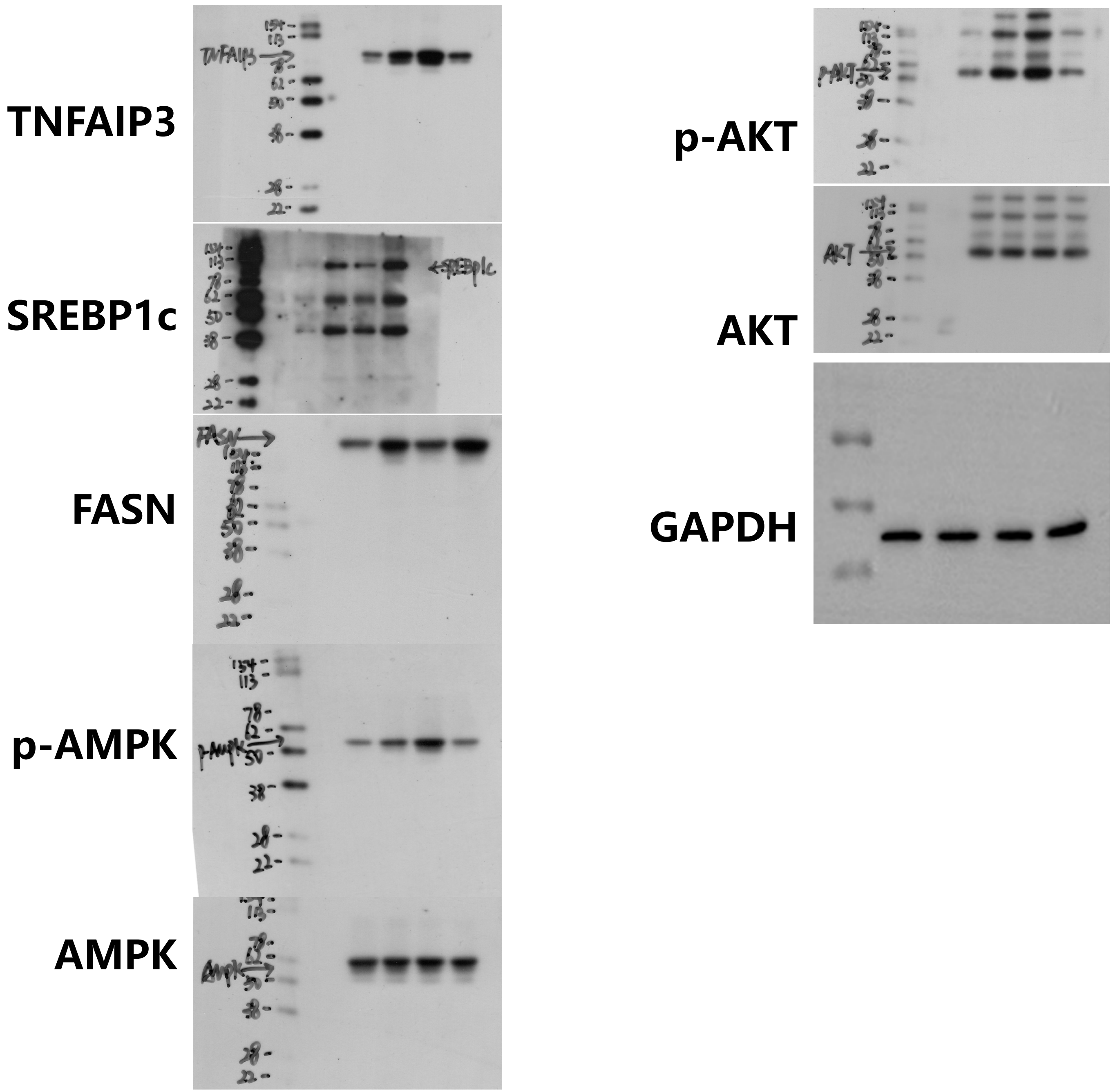

## Figure 3B:

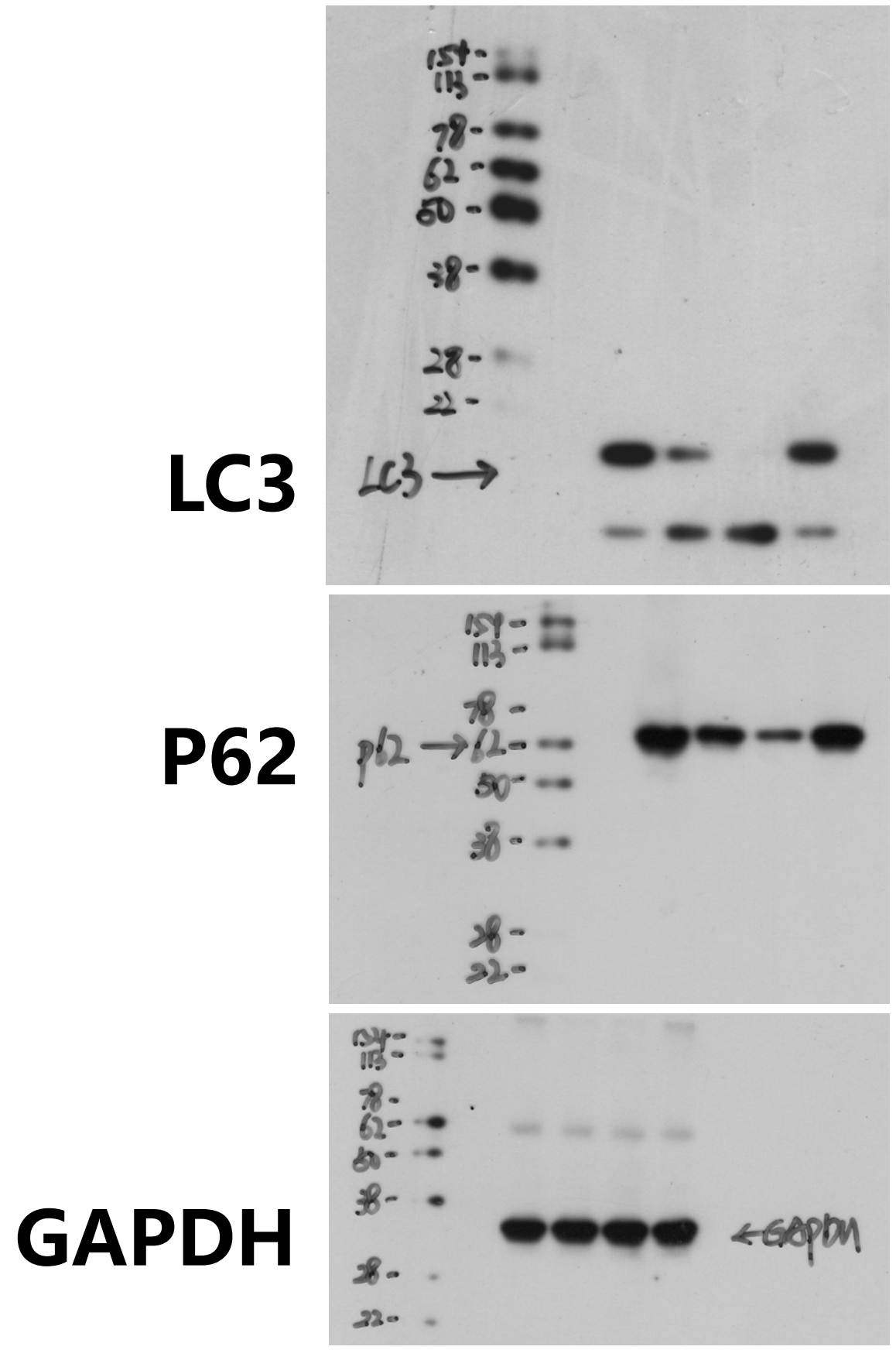

## Figure 3F:

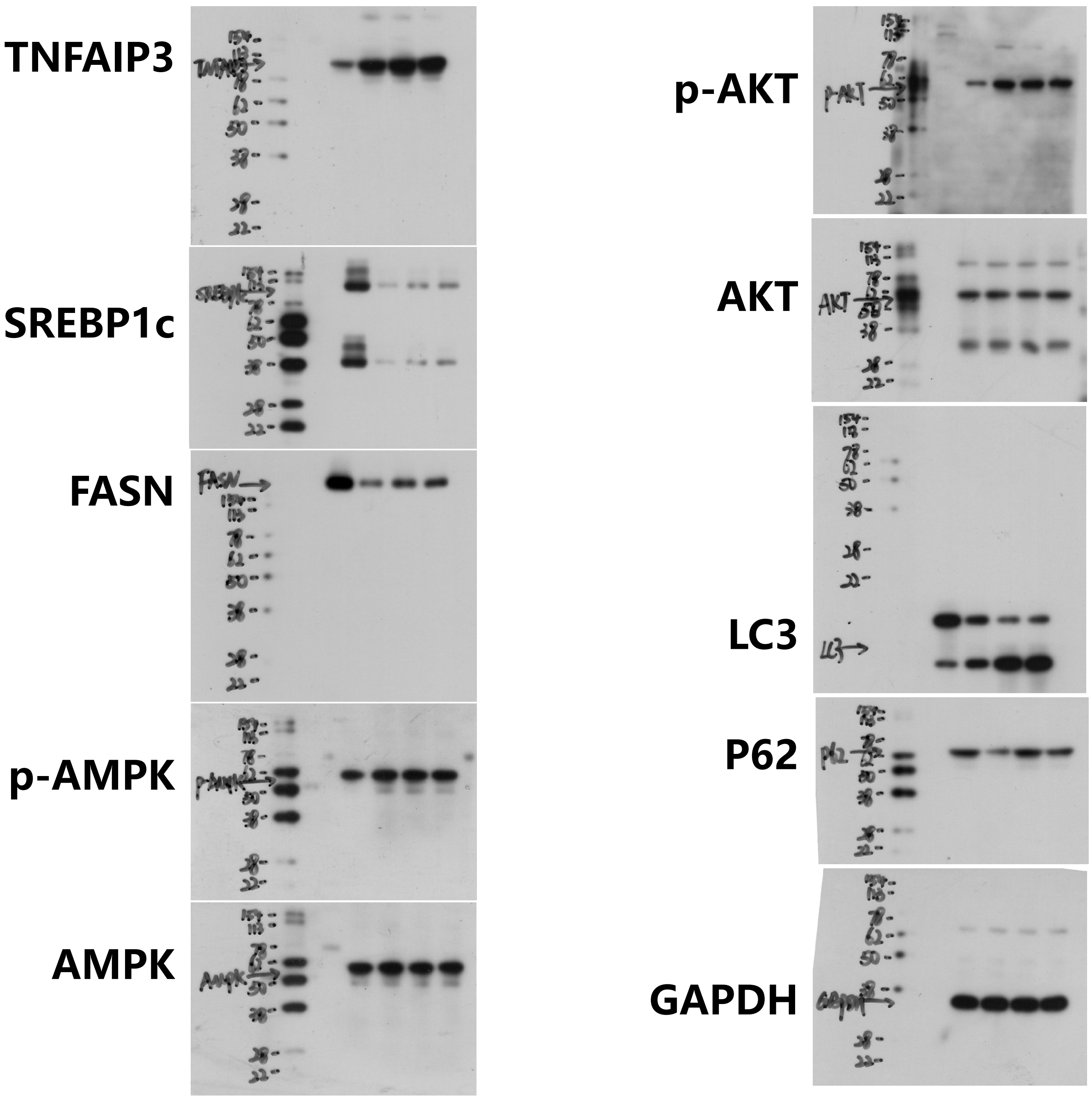

## Figure 5B:

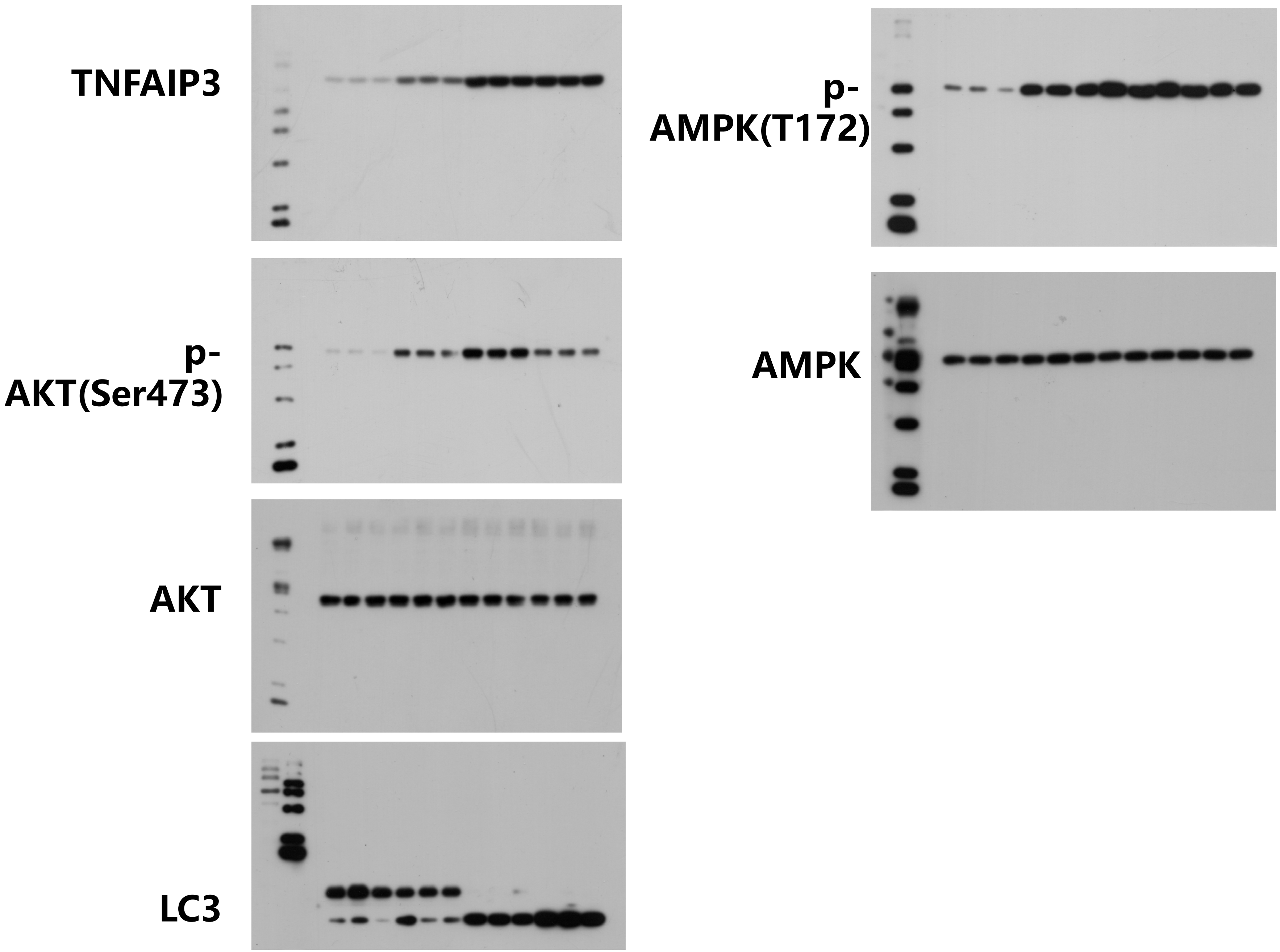

**Figure 5B:**

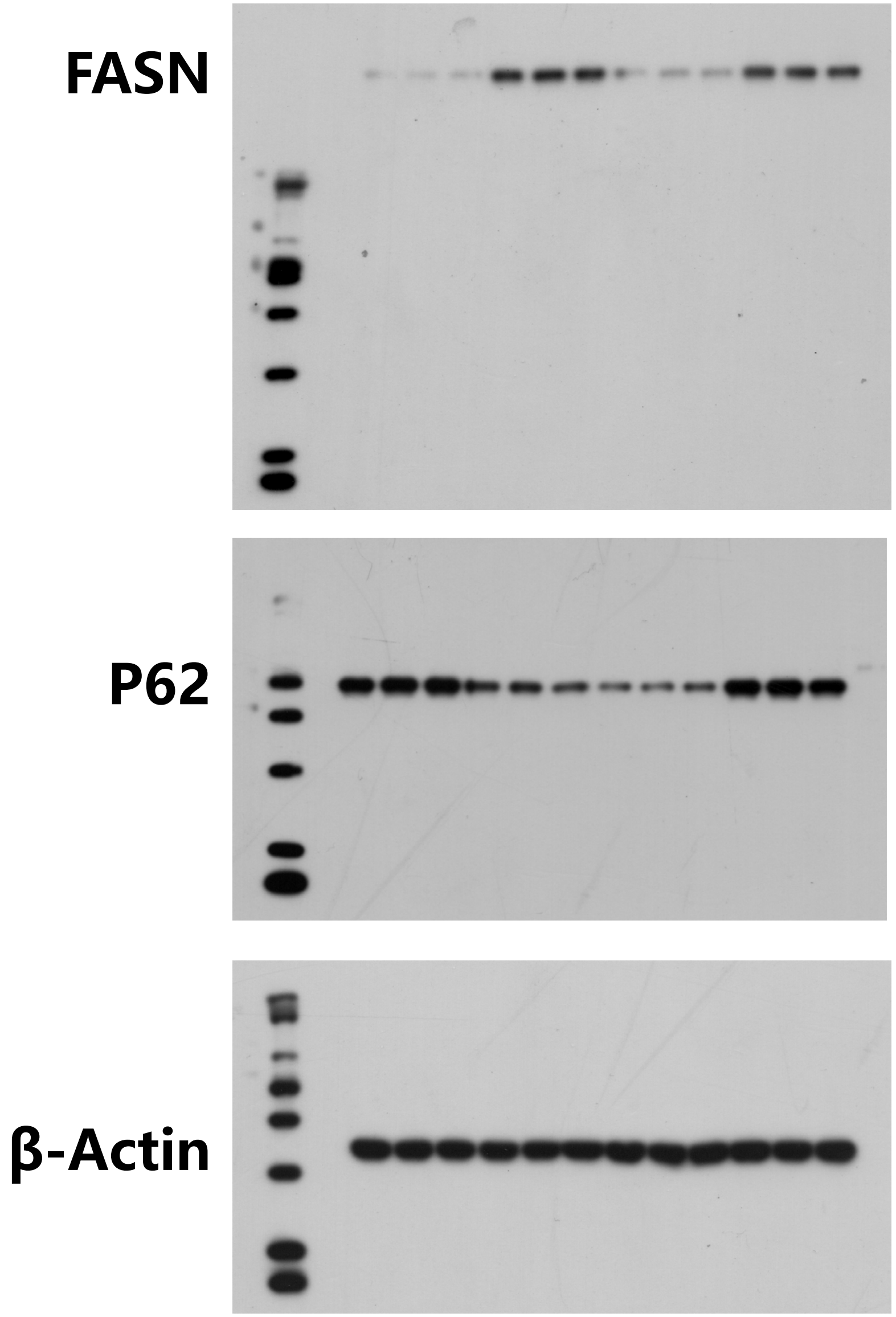

## Figure 5B:

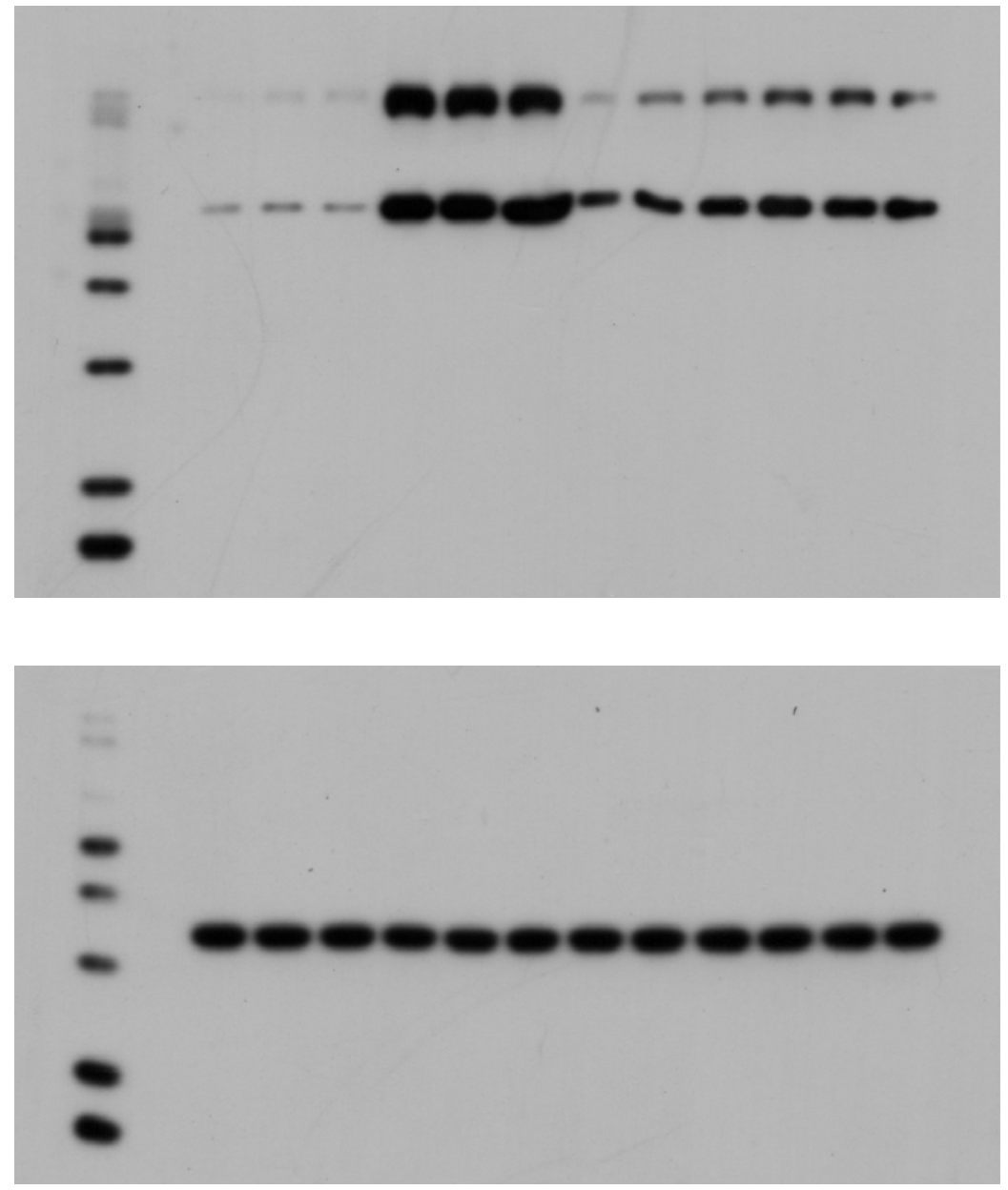

**Figure 6D:**

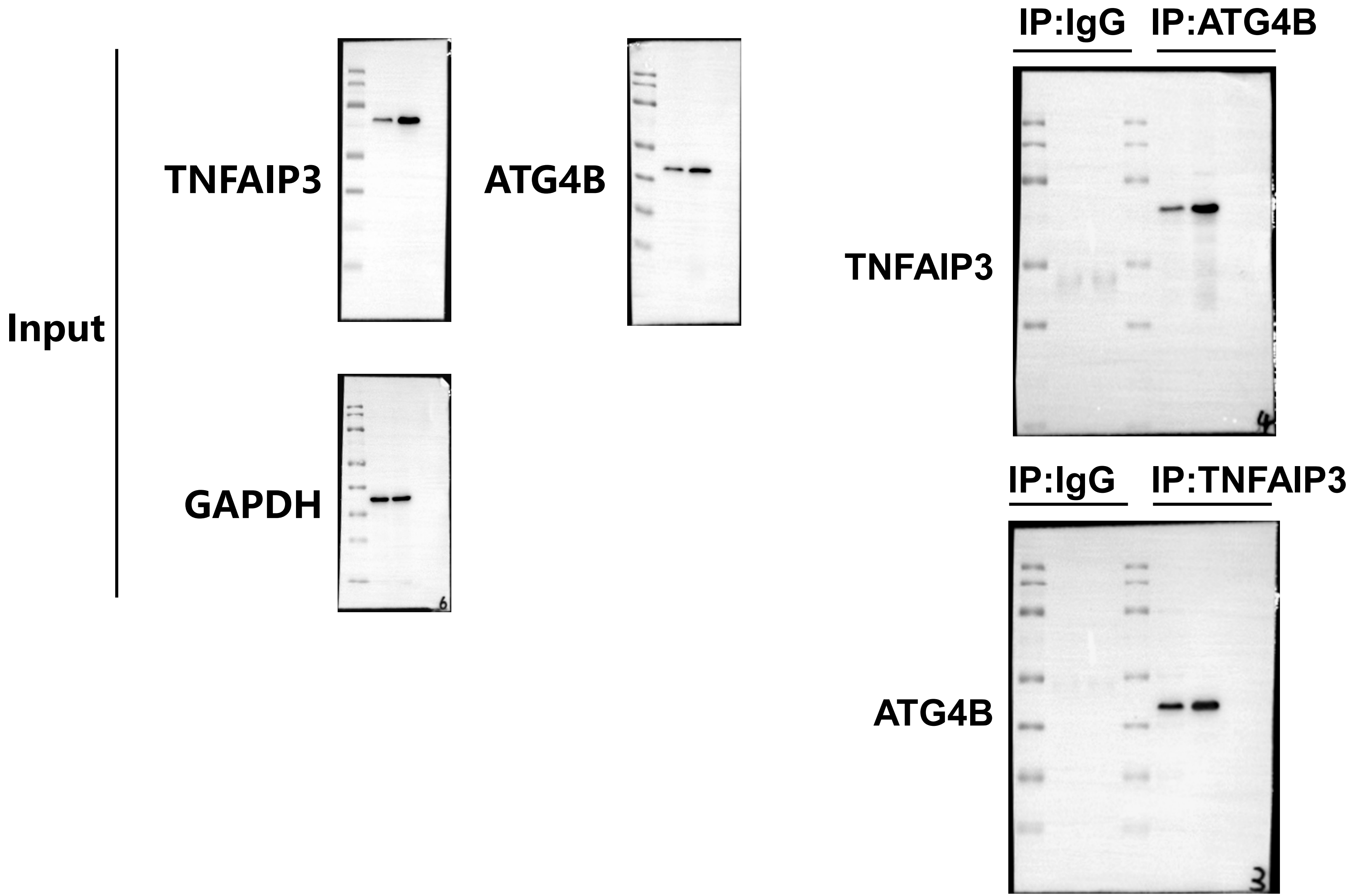
Input
TNFAIP3
ATG4B
GAPDH
IP:IgG
IP:ATG4B
TNFAIP3
IP:IgG
IP:TNFAIP3
ATG4B

**Figure 6E:**

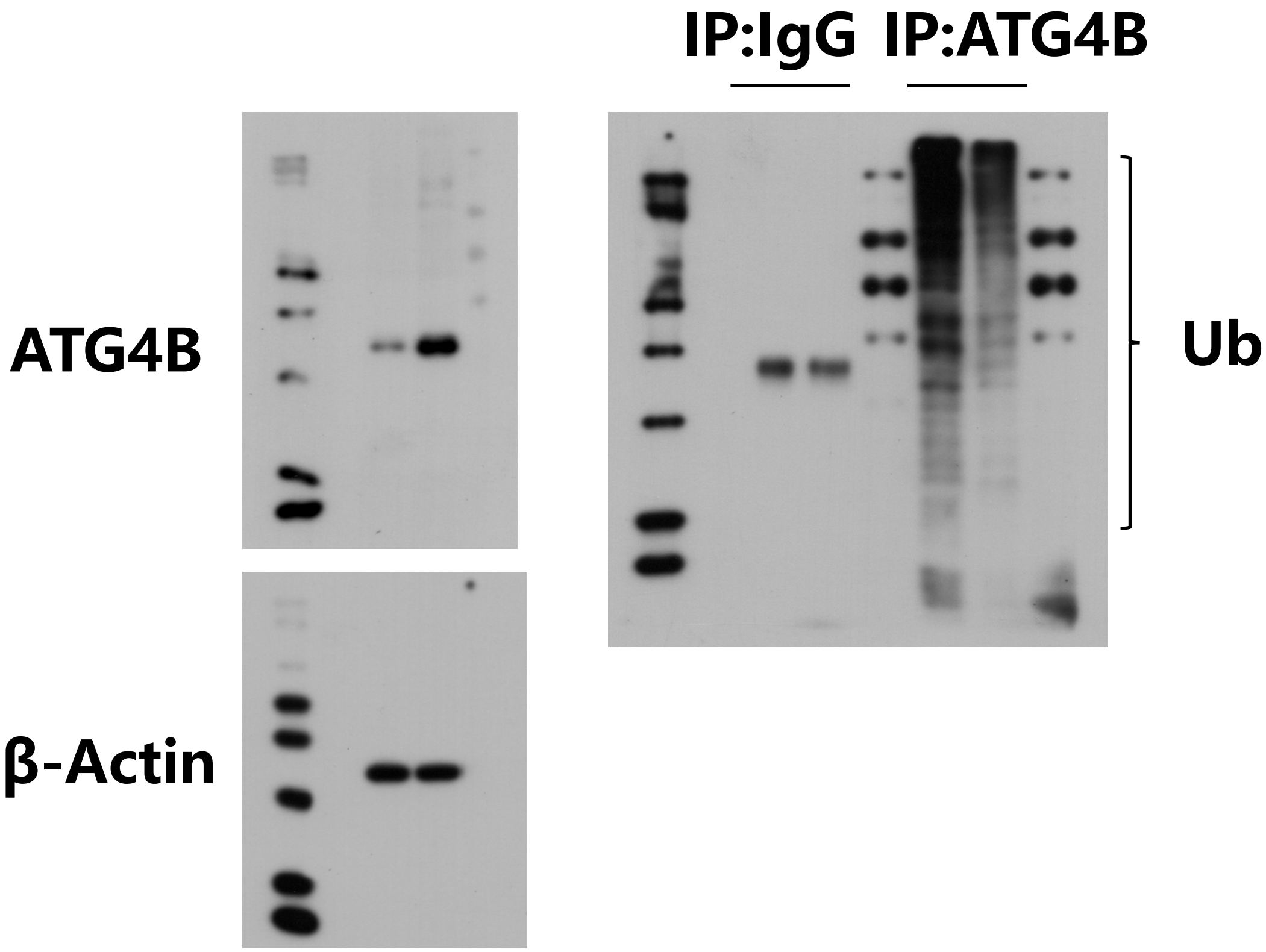

**Figure 6F:**

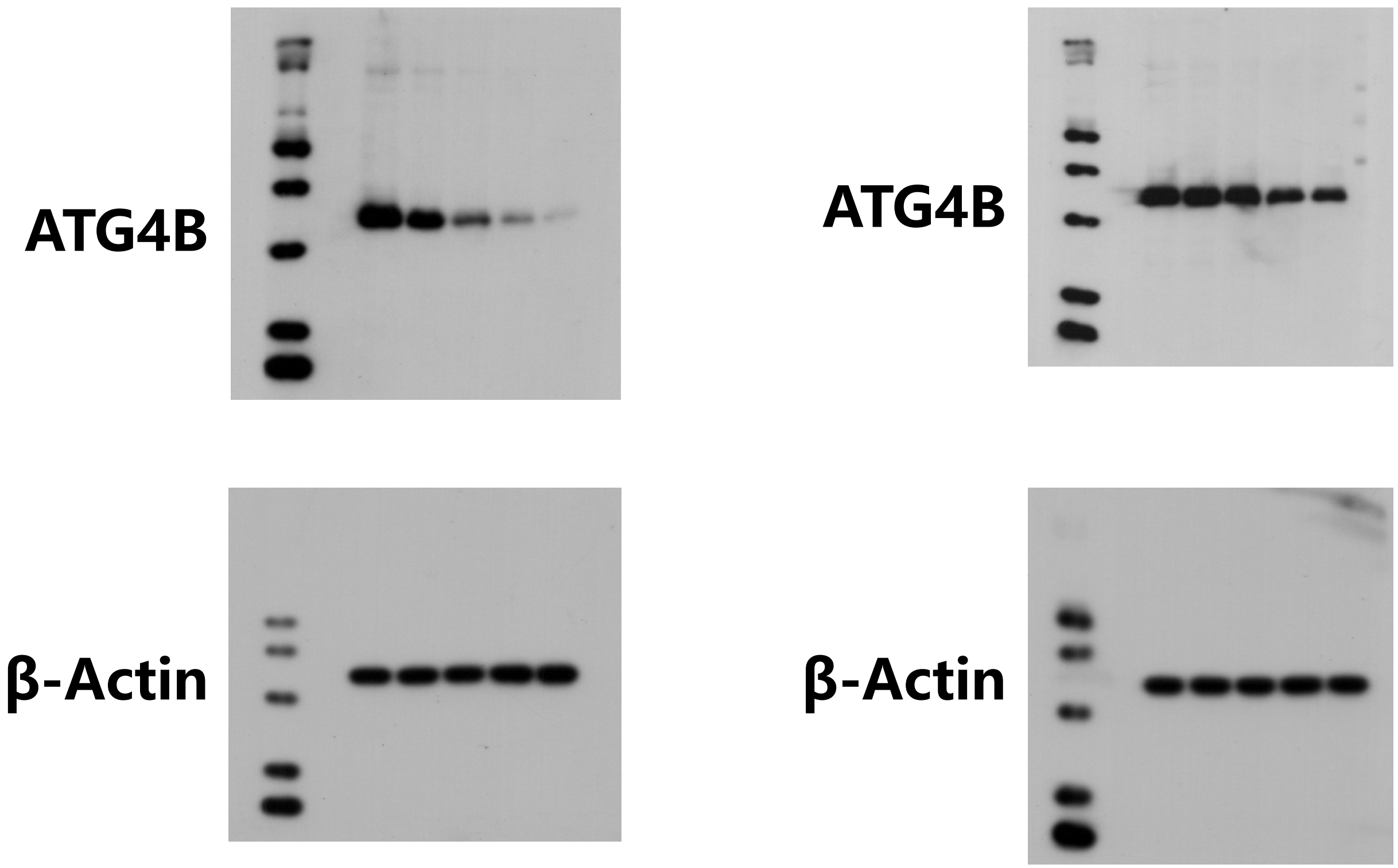

## Figure 6G:

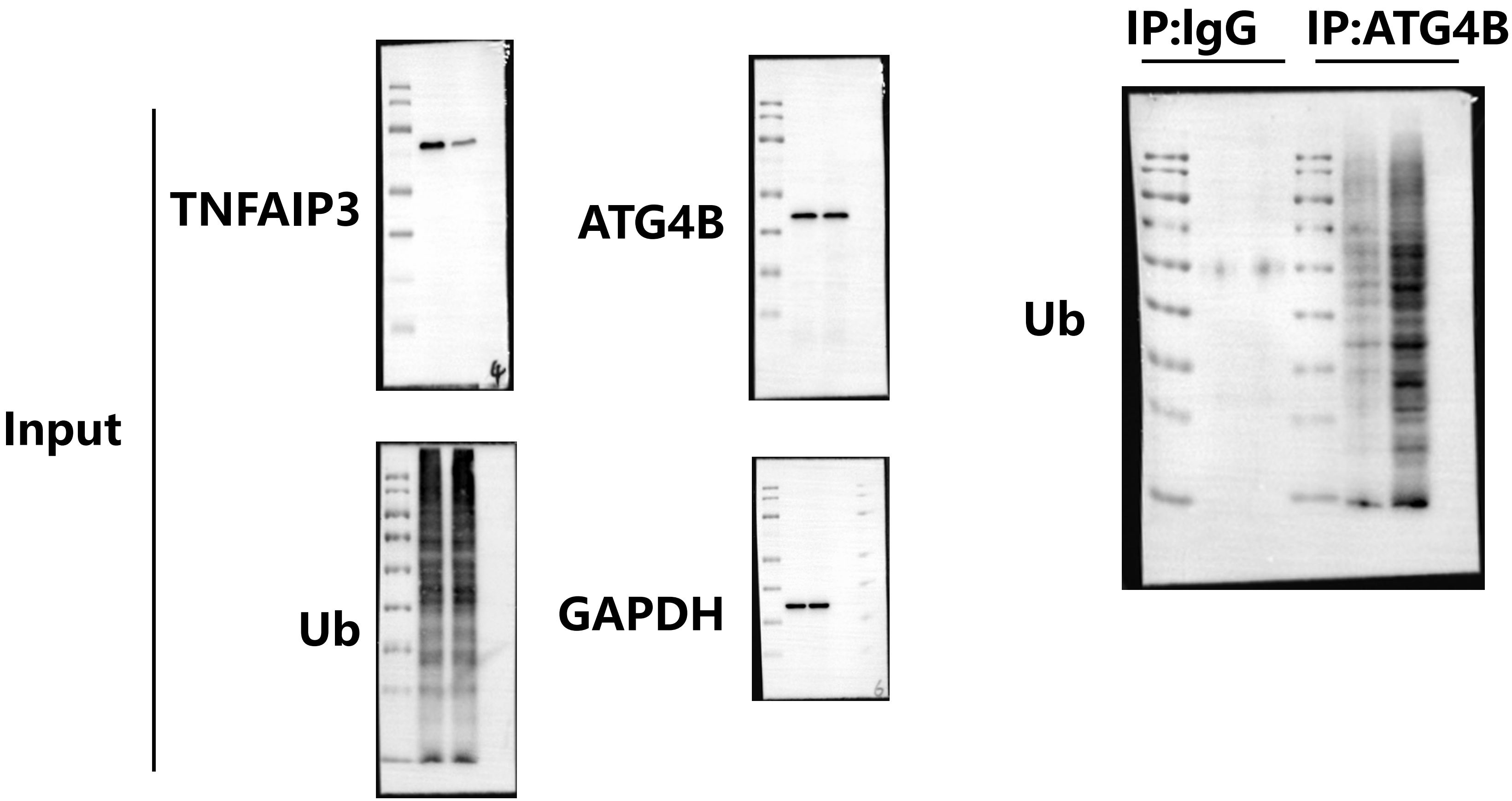

## Figure 7E:

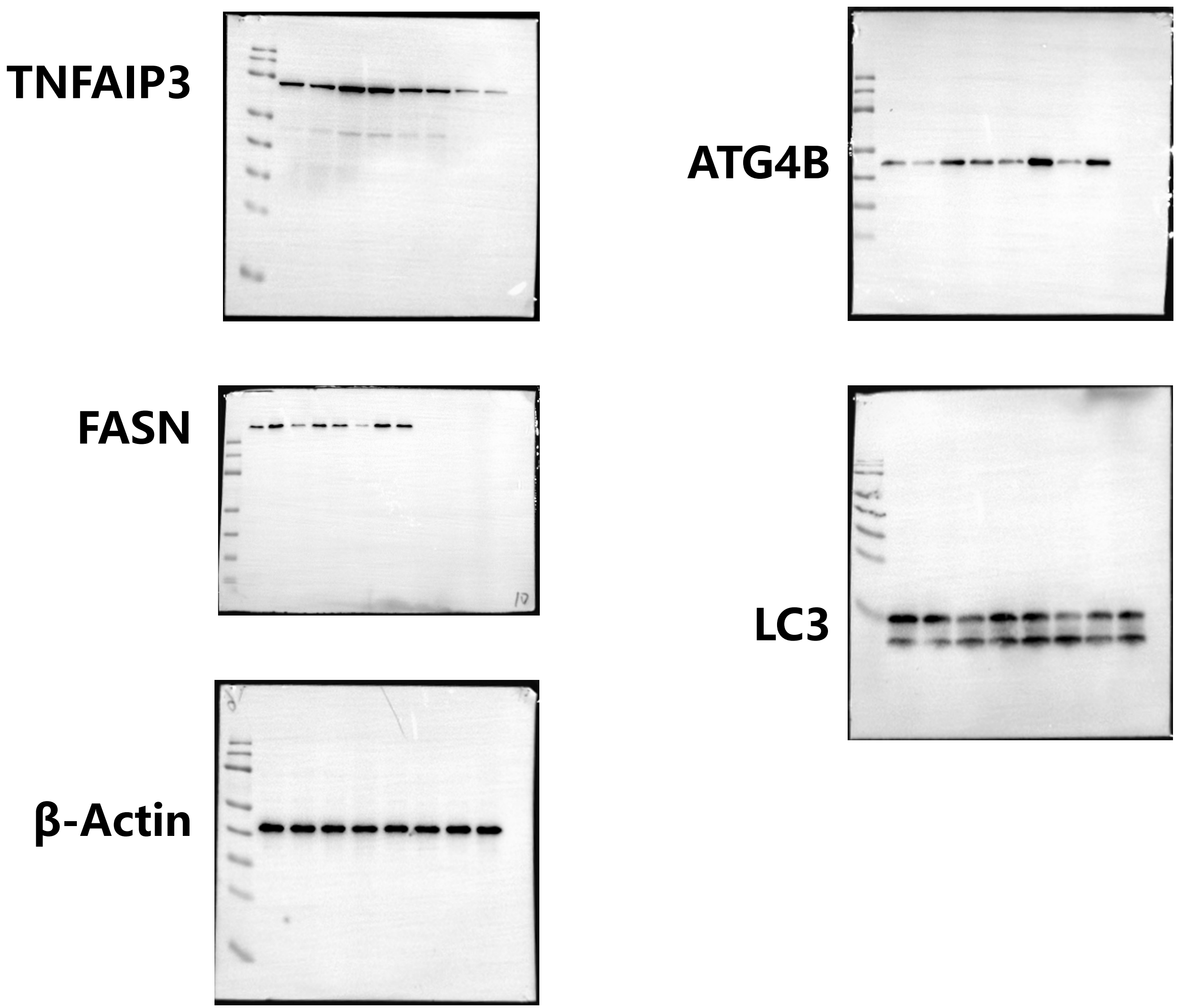

## Figure 8C:

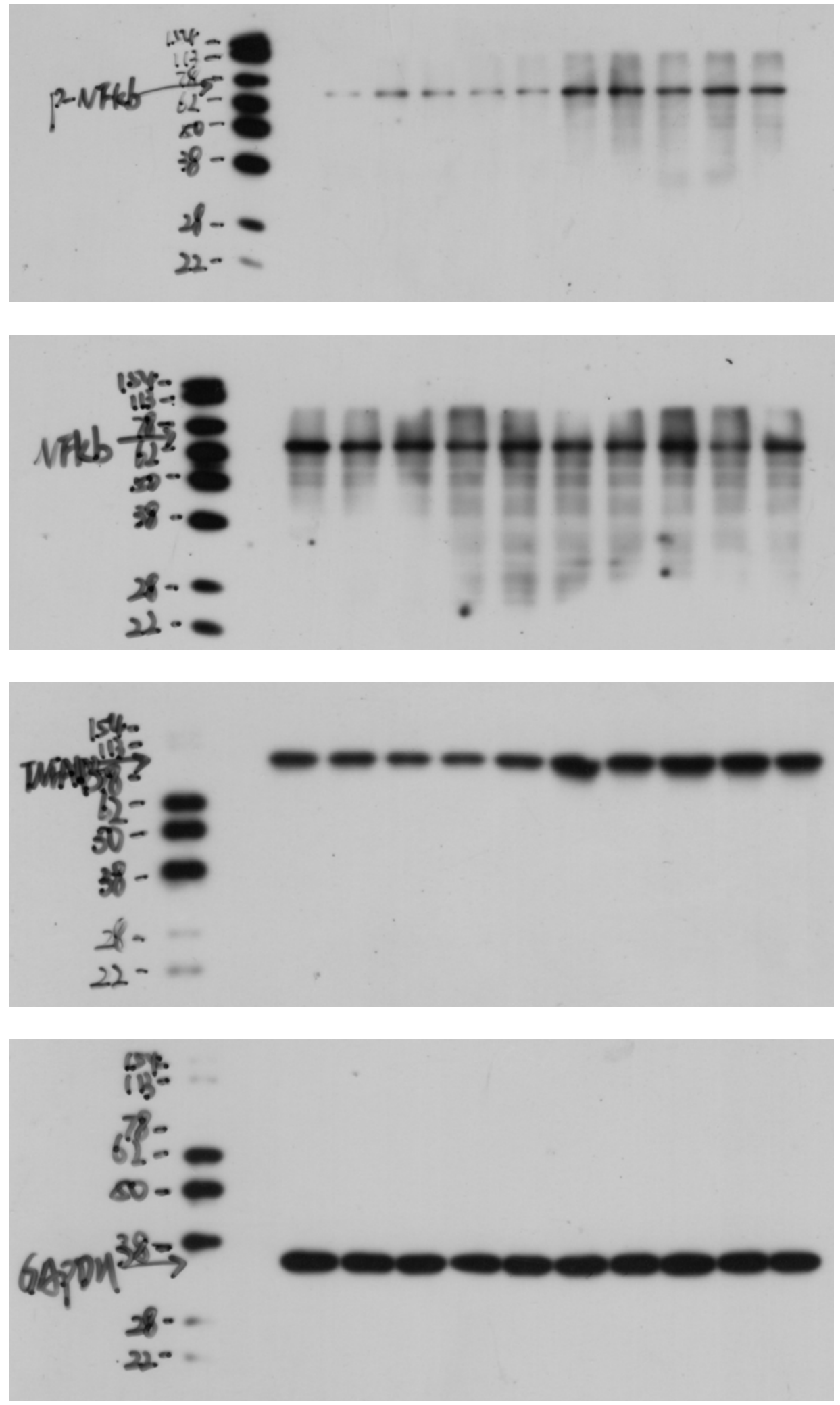

p-p65

p65

TNFAIP3

GAPDH

## Figure 8D:

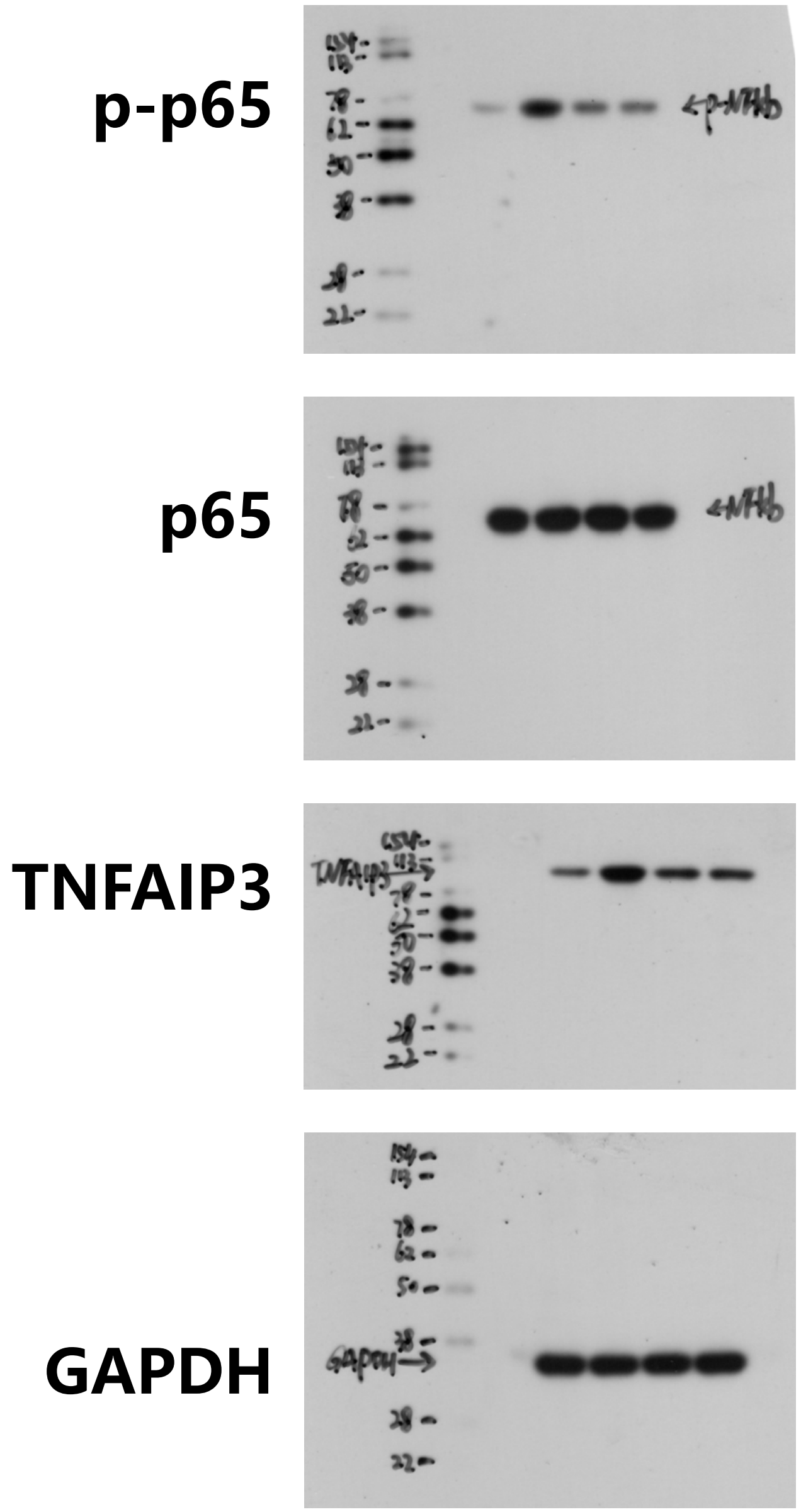

## Figure S9:

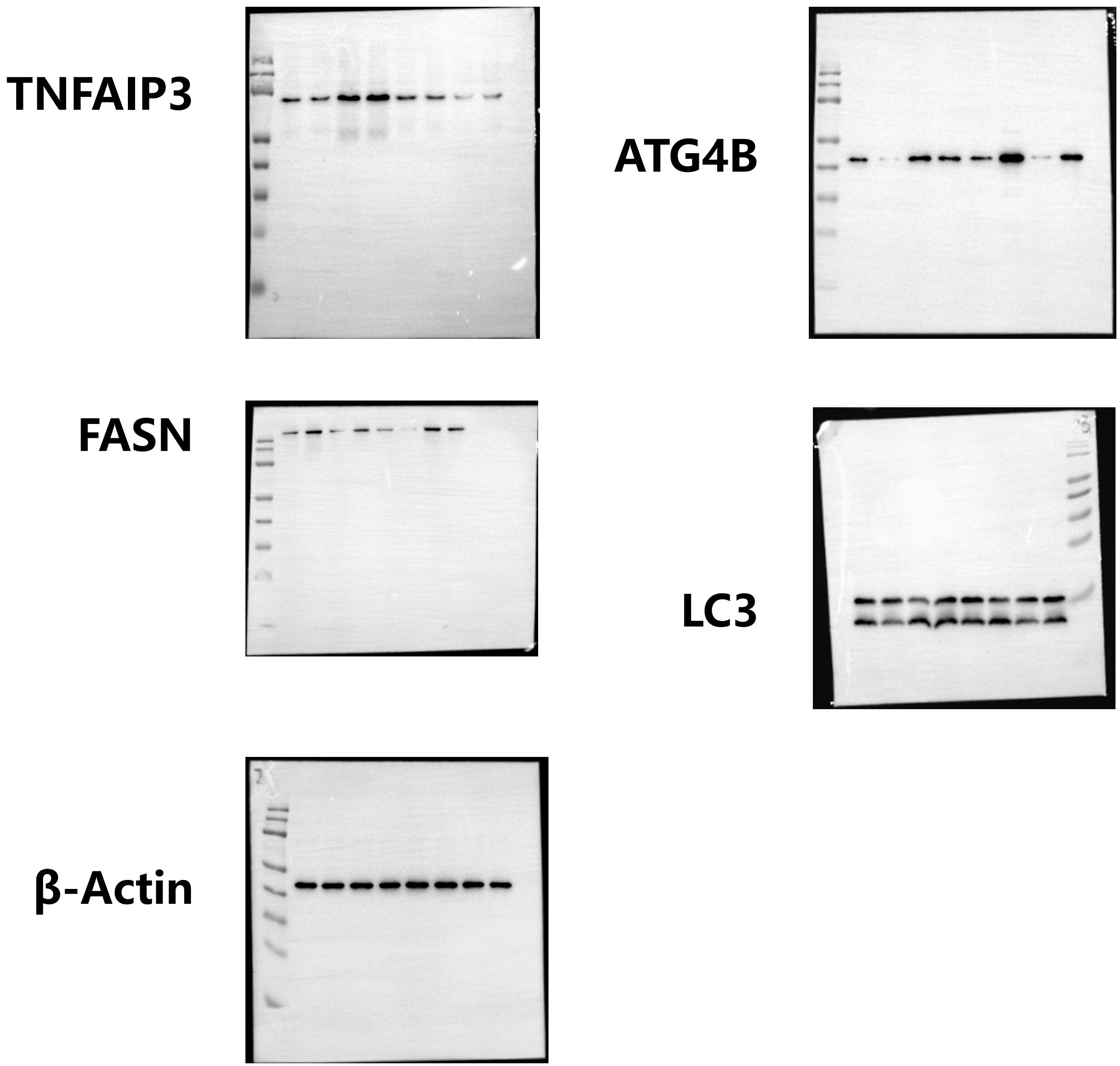

Supplement: Supplementary file 1 [file 41420_2026_3310_MOESM2_ESM.pdf]
