# Supplementary material for: The NF-κB-TNFAIP3 axis attenuates MASLD via activation of hepatocyte autophagy
Source: Cell Death Discov. 2026 Aug 25;12:430. doi: 10.1038/s41420-026-03310-9 (PMC13720041; doi:10.1038/s41420-026-03310-9)
Supplement: Supplementary file 2 [file 41420_2026_3310_MOESM1_ESM.docx]

**Supplementary materials**

**The NF-κB-TNFAIP3 Axis Attenuates MASLD via Activation of Hepatocyte Autophagy**

| **Clinical feature** | **Control** | | | **Hepatic steatosis** | | |
| --- | --- | --- | --- | --- | --- | --- |
| Patient No. | 1 | 2 | 3 | 1 | 2 | 3 |
| Age (years) | 68 | 71 | 68 | 71 | 37 | 57 |
| Sex (% female) | M | M | M | F | F | M |
| Body weight (kg) | 47 | 65.7 | 76 | 58 | 72.5 | 65 |
| Body mass index | 18.8 | 24.6 | 25.7 | 23.2 | 28.3 | 23.3 |
| Fasting blood glucose | 4.48 | 8.26 | 3.51 | 5.5 | 4.79 | 5.8 |
| T2DM* | N | Y | N | N | N | N |
| Platelets (×10^9^/L) | 156 | 141 | 133 | 127 | 167 | 169 |
| Albumin (g/dL) | 37.9 | 42.6 | 38.9 | 39 | 50.1 | 48 |
| ALT (U/L) | 14 | 41 | 9 | 21 | 71 | 52 |
| AST (U/L) | 22 | 37 | 24 | 39 | 53 | 42 |
| ALP (U/L) | 61 | 179 | 88 | 87 | 77 | 74 |
| GGT (U/L) | 26 | 327 | 54 | 16 | 72 | 35 |
| Serum bilirubin（μmol/L） | 9.9 | 12.4 | 10.5 | 18 | 22 | 42 |
| Serum creatinine（μmol/L） | 78.3 | 89.8 | 74.8 | 66 | 48 | 74 |
| INR | 0.96 | 1.09 | 0.91 | 1.06 | 0.95 | 1.02 |
| Alcohol assumption* | Y | Y | N | N | N | N |
| Smoking* | Y | N | N | N | N | N |
| Hypertension* | N | Y | Y | Y | N | Y |
| Steatosis | 0 | 0 | 0 | 3 | 3 | 2 |
| Ballooning | 1 | 0 | 0 | 2 | 0 | 1 |
| Lobular inflammation | 1 | 2 | 1 | 1 | 0 | 1 |
| Fibrosis | 0 | 0 | 0 | 1a | 1b | 1b |

**Table S1. Baseline characteristics of human liver tissue donors**

* Y = patient has a previous or personal history of the condition; N = patient does not have the condition.

**Table S2. Primary antibodies used in this study.**

| Antibody | Molecular Weight (kDa) | Host | Concentration | Company | Catalog No. |
| --- | --- | --- | --- | --- | --- |
| **Human livers/HepG2 cells** | | | | | |
| TNFAIP3 | 90 | Rabbit | 1/1000 | Abcam | ab92324 |
| TNFAIP3 | 90 | Mouse | 1/500 | Proteintech | 66695-1-Ig |
| p-AMPK(Thr172) | 62 | Rabbit | 1/1000 | CST | 2535 |
| AMPK | 62 | Rabbit | 1/1000 | CST | 5831 |
| p-AKT(Ser473) | ~60 | Mouse | 1/1000 | Proteintech | 66444-1-IG |
| AKT | ~60 | Rabbit | 1/1000 | Proteintech | 10176-2-AP |
| SREBP-1 | 125, 65 | Mouse | 1/1000 | Santa cruz | SC-365513 |
| FASN | 270 | Rabbit | 1/1000 | Abcam | ab128856 |
| p62 | 62 | Rabbit | 1/1000 | Servicebio | GB11239-1 |
| LC3 | 14, 16 | Rabbit | 1/1000 | Zen bio | 350140 |
| Phospho-NF-κB p65 | 65 | Rabbit | 1:1000 | CST | 3033T |
| NF-κB p65 | 65 | Rabbit | 1:1000 | Proteintech | 10745-1-AP |
| GAPDH | 36 | Rabbit | 1/20000 | CST | 5174 |
| ATG4B | 44 | Rabbit | 1/500~1/3000 | Proteintech | 15131-1-AP |
| Ubiquitin (Ub) | / | Rabbit | 1/500~1/1000 | Proteintech | 10201-2-AP |
| β-Actin | 42 | Mouse | 1/20000~1/100000 | Proteintech | 66009-1-Ig |
| **Mouse livers/Primary mouse hepatocytes** | | | | | |
| TNFAIP3 | 90 | Rabbit | 1/1000~1/5000 | Abcam | ab92324 |
| p-AMPK(Thr172) | 62 | Rabbit | 1/1000 | CST | 2535 |
| AMPK | 62 | Rabbit | 1/1000 | CST | 5831 |
| p-AKT(Ser473) | ~60 | Rabbit | 1/5000~1/50000 | Proteintech | 80455-1-RR |
| AKT | ~60 | Rabbit | 1/2000~1/12000 | Proteintech | 10176-2-AP |
| SREBP1 | 125, 65 | Rabbit | 1/500~1/5000 | Abcam | ab269577 |
| FASN | 270 | Rabbit | 1/10000 | Abcam | ab128870 |
| p62 | 62 | Rabbit | 1/10000~1/50000 | Abcam | ab109012 |
| LC3 | 14, 16 | Rabbit | 1/2000 | Abcam | ab192890 |
| LC3 | 14, 16 | Rabbit | 1/1000 | Selleck | F0144 |
| β-Actin | 42 | Mouse | 1/20000~1/100000 | Proteintech | 66009-1-Ig |
| Phospho-NF-κB p65 | 65 | Rabbit | 1:1000 | CST | 3033T |
| NF-κB p65 | 65 | Rabbit | 1:1000 | Proteintech | 10745-1-AP |
| GAPDH | 36 | Rabbit | 1/20000 | CST | 5174 |

**Table S3. Characteristics of GEO datasets analyzed in this study*.**

| ID | Year | Overall design | Sample | Platform |
| --- | --- | --- | --- | --- |
| GSE256063 | 2025 | Chow diet: n=8; AMLN diet: n=8 | Liver | GPL19057 |
| GSE246328 | 2024 | C57BL/6J mice: Chow diet for 24 weeks: n=8; GAN diet for 24 weeks: n=7 | Liver | GPL19057 |
| GSE256501 | 2024 | C57BL/6J mice: Chow diet for 16 weeks: n=6; FPC diet for 16 weeks: n=6. | Liver | GPL17021 |
| GSE256502 | 2024 | C57BL/6J mice: Chow diet for 16 weeks: n=8; FPC diet for 16 weeks: n=8. | Hepatocyte | GPL17021 |

*All datasets were derived from Mus musculus and involved RNA sequencing.

**Table S4. Differential proteins identified by TNFAIP3** **immunoprecipitation**-**mass spectrometry in HepG2 cells**

| Accession number | Gene | Coverage (%) | Unique peptides | -10lgP |
| --- | --- | --- | --- | --- |
| Q58FF7 | HSP90AB3P | 45 | 1 | 322.81 |
| P01023 | A2M | 40 | 41 | 306.58 |
| P05023 | ATP1A1 | 43 | 30 | 305.2 |
| P13674 | P4HA1 | 66 | 32 | 302.73 |
| P0C0L5 | C4B_2 | 32 | 2 | 299.86 |
| Q01518 | CAP1 | 77 | 36 | 298.62 |
| P27797 | CALR | 73 | 35 | 295.71 |
| P54577 | YARS1 | 77 | 41 | 293.13 |
| Q02790 | FKBP4 | 75 | 39 | 289.97 |
| P40926 | MDH2 | 70 | 21 | 284.15 |

| **ID** | **Position** | **Code** | **E3 enzymes** | **Peptide** | **Score** | **Cutoff** |
| --- | --- | --- | --- | --- | --- | --- |
| NP_037457.3 ATG4B isoform a | 39 | K | General | LGRKYSIFTEKDEILSDVASR | 0.4995 | 0.2655 |
| NP_037457.3 ATG4B isoform a | 56 | K | General | VASRLWFTYRKNFPAIGGTGP | 0.3989 | 0.2655 |
| NP_037457.3 ATG4B isoform a | 119 | K | General | FSVLNAFIDRKDSYYSIHQIA | 0.6537 | 0.2655 |
| NP_037457.3 ATG4B isoform a | 153 | K | General | YGPNTVAQVLKKLAVFDTWSS | 0.5878 | 0.2655 |
| NP_037457.3 ATG4B isoform a | 154 | K | General | GPNTVAQVLKKLAVFDTWSSL | 0.5164 | 0.2655 |
| NP_037457.3 ATG4B isoform a | 337 | K | General | DDFNDWCQQVKKLSLLGGALP | 0.3498 | 0.2655 |

**Table S5. Predicted ubiquitination sites of ATG4B in Homo sapiens using the GPS**-**Uber database**

**Figure S1. TNFAIP3 is upregulated in the liver across distinct diet**-**induced MASLD mouse models.** (A) Volcano plots illustrating differentially expressed genes in the liver of mice fed an AMLN or GAN diet, compared with their respective controls. (B) Heatmap showing hepatic expression of genes involved in lipogenesis, inflammation, and lipid regulation in mice fed an AMLN or GAN diet

**Figure S2. TNFAIP3 protects against lipid accumulation in hepatocytes.** (A) TNFAIP3 mRNA levels measured by RT-qPCR in HepG2 cells transfected with TNFAIP3-targeting shRNA or control shRNA. (B) Representative Oil Red O staining of PA-treated HepG2 cells with TNFAIP3 overexpression or knockdown.

**Figure S3. TNFAIP3 enhances autophagic activity in hepatocytes.** (A-C) Gene Ontology (GO) enrichment analysis of differentially expressed genes between high- and low- TNFAIP3 expression groups in livers from FPC- (A), AMLN- (B) or GAN- (C) diet-induced MASLD mouse models. (D) Representative Oil Red O staining of PA-treated HepG2 cells overexpressing TNFAIP3, with or without co-treatment with the autophagy inhibitors bafilomycin A1 (BafA1) or chloroquine (CQ).

**Figure S4. TNFAIP3 alleviates MASLD**-**related hepatic pathology in an autophagy**-**dependent manner in vivo.** (A-C) NAFLD activity score (A), relative area of lipid droplets, and collagen volume fraction in liver sections from HF diet-fed mice with TNFAIP3 overexpression, with or without CQ treatment. (D) Quantification of TNFAIP3-positive area by immunohistochemistry in liver tissues from HF diet-fed mice and controls. **P* < 0.05; ****P* < 0.001; *****P* < 0.0001.

**Figure S5. TNFAIP3 improves glucose homeostasis via autophagy in vivo.** (A-B) Fasting glucose levels and area under the curve (AUC) values derived from glucose tolerance test (GTT) (A) and insulin tolerance test (ITT) (B) of HF diet-fed mice with TNFAIP3 overexpression, with or without CQ treatment. ****P* < 0.001; *****P* < 0.0001.

**Figure S6. Tandem mRFP-GFP-LC3 fluorescence assay in PA**-**treated HepG2 cells subjected to TNFAIP3 overexpression, ATG4B knockdown, or combined TNFAIP3 overexpression and ATG4B knockdown.** ** and *** indicate *P* < 0.01 and *P* < 0.001, respectively, compared with the PA group. &&&& indicates *P* < 0.0001 compared with the PA+TNFAIP3-OE group. # and ## indicate *P* < 0.05 and *P* < 0.01, respectively, compared with the PA+siATG4B group.

**Figure S7. Top 20 GO biological process terms enriched among TNFAIP3**-**interacting proteins.**
**Figure S8. ATG4B mRNA levels measured by RT-qPCR in primary mouse hepatocytes and HepG2 cells transfected with ATG4B**-**targeting shRNA or control shRNA.** ***P* < 0.01; *****P* < 0.0001.

**Figure S9. TNFAIP3**-**ATG4B axis modulates hepatic lipid accumulation.** (A, C) Representative immunofluorescence images of lipid droplets (A) and intracellular TC and TG levels (C) in PA-treated HepG2 cells subjected to ATG4B knockdown, TNFAIP3 overexpression, or combined TNFAIP3 overexpression and ATG4B knockdown. (B, D) Representative immunofluorescence images of lipid droplets (B) and intracellular TC and TG levels (D) in PA-treated HepG2 cells subjected to ATG4B overexpression, TNFAIP3 knockdown, or combined TNFAIP3 knockdown and ATG4B overexpression. (E) Western blot analysis of TNFAIP3, ATG4B, LC3, and FASN expression in PA-treated HepG2 cells following manipulation of TNFAIP3 and ATG4B expression. **P* < 0.05; ****P* < 0.001; *****P* < 0.0001; n.s., non-significant.

**Figure S10. Relative luciferase activity of TNFAIP3 promoter following treatment with NF**-**κB inhibitors JSH**-**23 or NF**-**κB**-**IN**-**1 in PA**-**treated HepG2 cells.** **P* < 0.05; ***P* < 0.01; ****P* < 0.001.
